# Supplementary figures and images for: Cortical representations of numbers and nonsymbolic quantities expand and segregate in children from 5 to 8 years of age
Source: PLoS Biol. 2023 Jan 5;21(1):e3001935. doi: 10.1371/journal.pbio.3001935 (PMC9815645; doi:10.1371/journal.pbio.3001935)

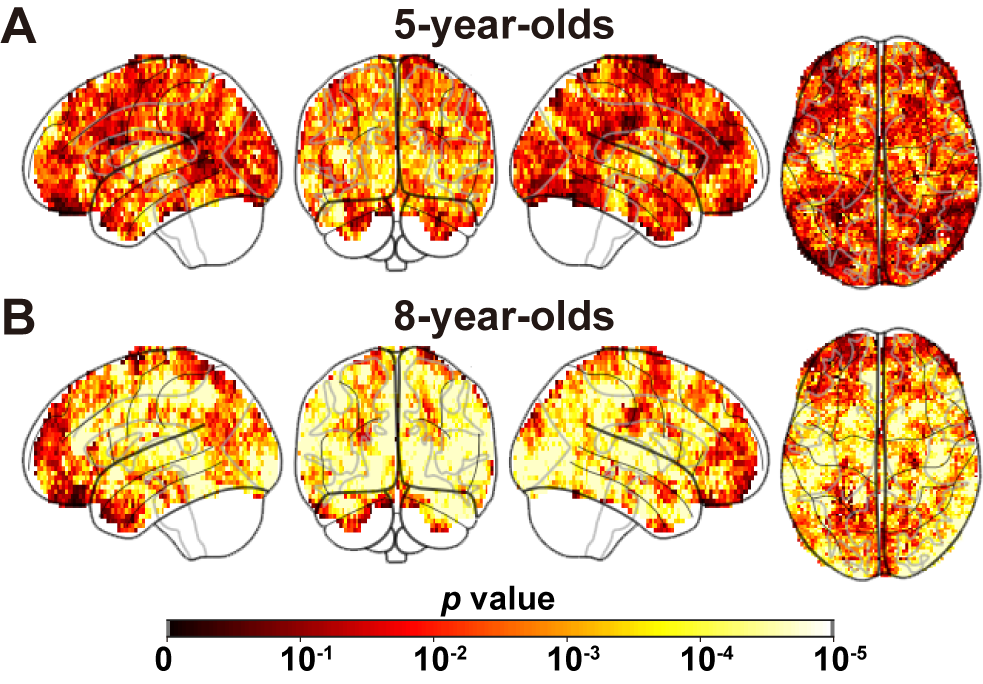

Supplement: S1 Fig — (A-B) Voxels in which activity could accurately classify between adaptation versus no-adaptation blocks of dots based on training with the same format, shown with unthresholded maps for (A) 5-year-olds and (B) 8-year-olds. The underlying data supporting this figure can be found online in file “Dots_[5yo/8yo]_LogPval.nii” in “UnthresholdData” folder. (TIF) [file pbio.3001935.s001.tif]

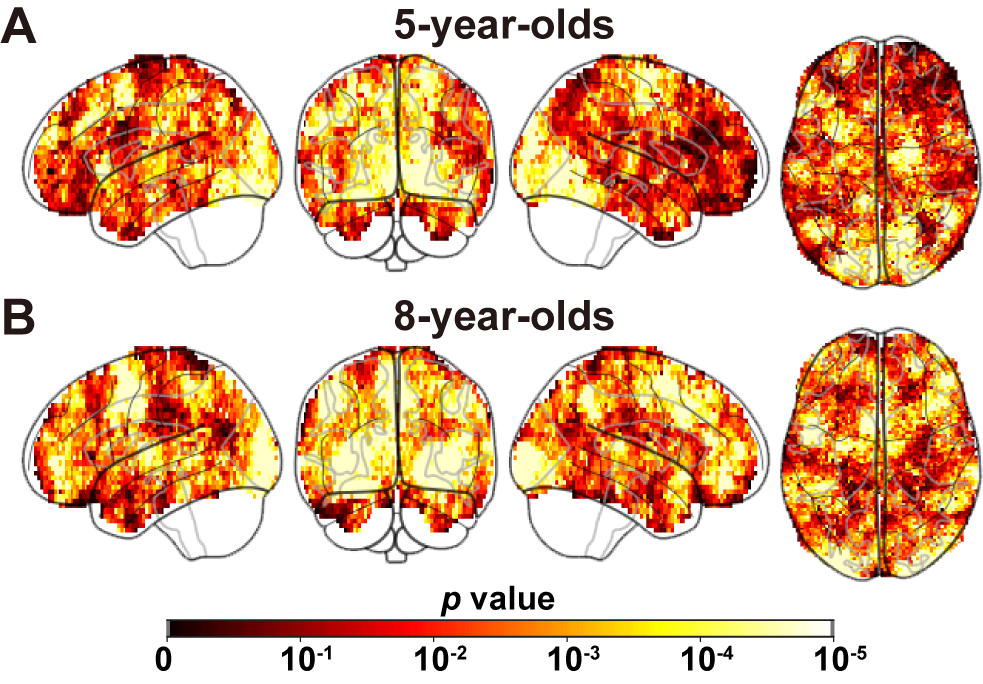

Supplement: S2 Fig — (A-B) Voxels in which activity could accurately classify between adaptation versus no-adaptation blocks of digits based on training with the same format, shown with unthresholded maps for (A) 5-year-olds and (B) 8-year-olds. The underlying data supporting this figure can be found online in files “Digits_[5yo/8yo]_LogPval.nii” in “UnthresholdData” folder. (TIF) [file pbio.3001935.s002.tif]

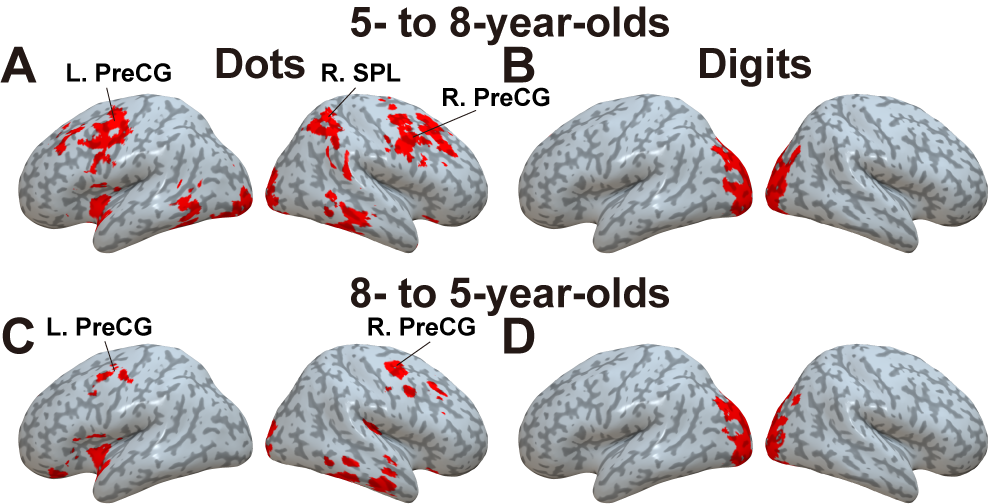

Supplement: S3 Fig — (A-B) Brain regions in which activity could accurately classify between adaptation versus no-adaptation blocks of (A) dots and (B) digits in 8-year-olds based on training with the same format in the 5-year-olds. (C-D) Brain regions in which activity could accurately classify between adaptation versus no-adaptation blocks of (C) dots and (D) digits in 5-year-olds based on training with the same format in the 8-year-olds. Only statistically significant clusters are shown (sign permutation test, voxel-level p < 0.005, cluster-level p < 0.05 with false discovery rate correction). PreCG, precentral gyrus; SPL, superior parietal lobule. The underlying data supporting this figure can be found online in files “RawDecAcc_LOOCV_[5to8yo/8to5yo]_[Dots/Digits]_[Subjects’ ID].nii” in “RawDecAcc” folder and in files “[Dots/Digits]_[5to8/8to5]_LogPval.nii” in “UnthresholdData” folder. (TIF) [file pbio.3001935.s003.tif]

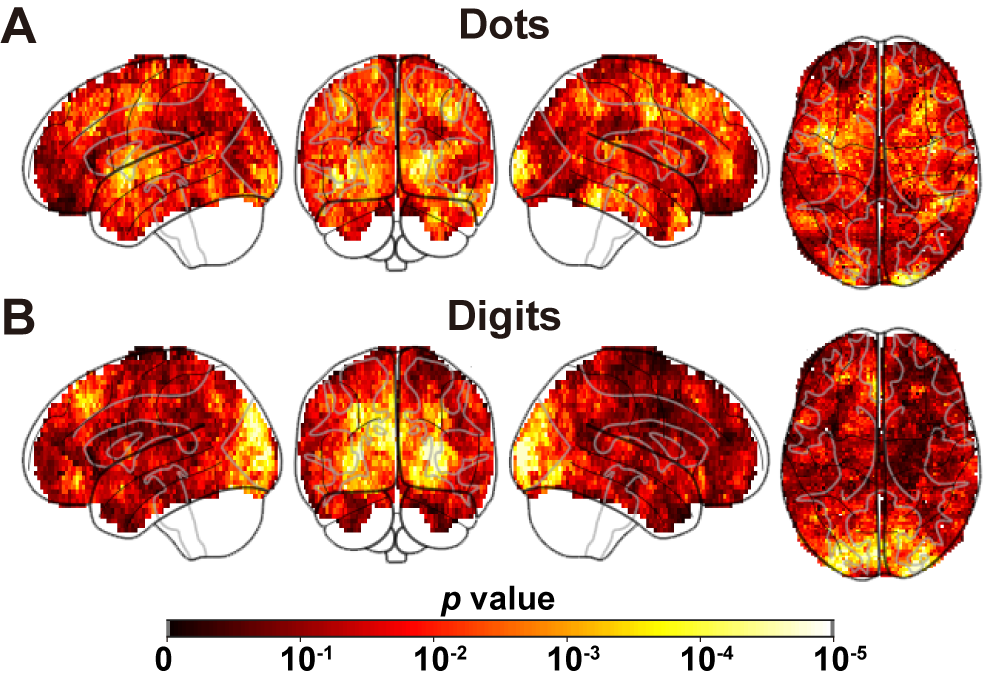

Supplement: S4 Fig — (A-B) Voxels in which activity could accurately classify between adaptation versus no-adaptation blocks of (A) dots and (B) digits in one group based on training with the same format in the other group (conjunction analysis), shown with unthresholded maps. The underlying data supporting this figure can be found online in files “[Dots/Digits]_5to8&8to5_LogPval.nii” in “UnthresholdData” folder. (TIF) [file pbio.3001935.s004.tif]

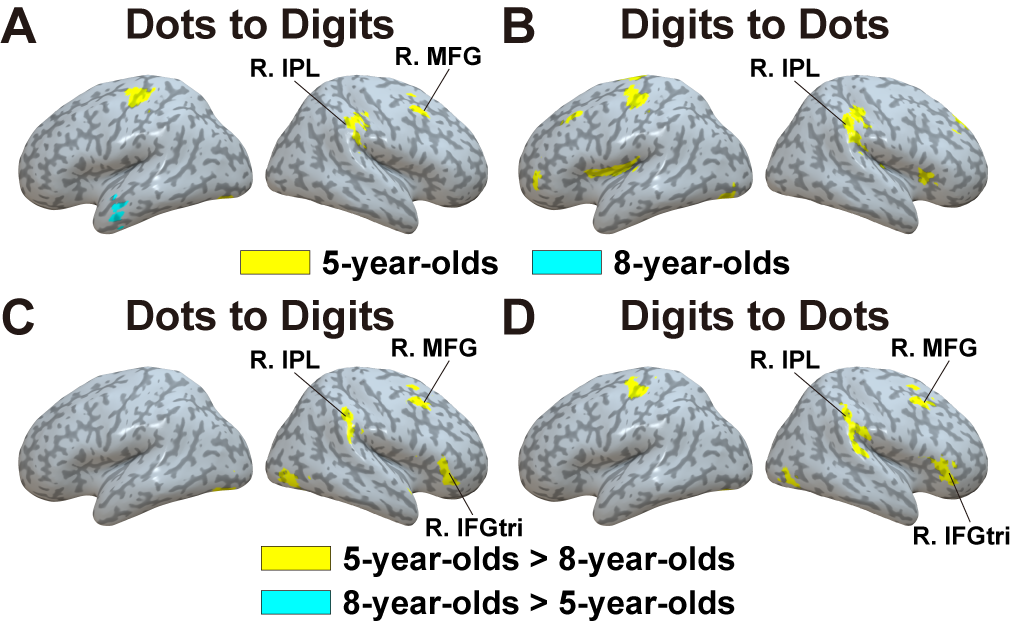

Supplement: S5 Fig — (A) Brain regions in which activity could accurately classify between adaptation versus no-adaptation blocks of digits based on training with dots. (B) Brain regions in which activity could accurately classify between adaptation versus no-adaptation blocks of dots based on training with digits. (C) Brain regions in which between-format decoding accuracy was larger in 5-year-olds than in 8-year-olds, tested with digits based on training with dots. (D) Brain regions in which between-format decoding accuracy was larger in 5-year-olds than in 8-year-olds, tested with dots based on training with digits. IFGtri, triangular part of inferior frontal gyrus; IPL, inferior parietal lobule; MFG, middle frontal gyrus; PreCG, precentral gyrus. The underlying data supporting this figure can be found online in files “RawDecAcc_LOOCV_[5yo/8yo]_[Dots2Digits/Digits2Dots]_[Subjects’ ID].nii” in “RawDecAcc” folder and in files “[Dots2Digits/Digits2Dots]_[5yo/8yo/5yo-8yo/8yo-5yo]_LogPval.nii” in “UnthresholdData” folder. (TIF) [file pbio.3001935.s005.tif]

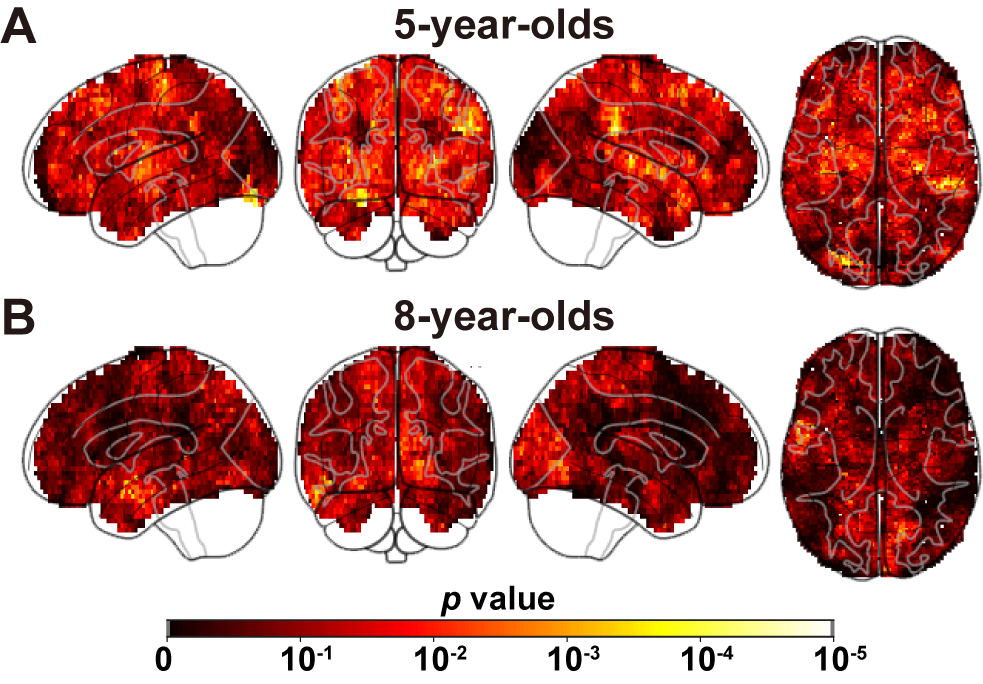

Supplement: S6 Fig — (A-B) Voxels in which activity could accurately classify between adaptation versus no-adaptation blocks of quantity in one format (dots or digits) based on training with the other format (digits or dots) (conjunction analysis), shown with unthresholded maps for (A) 5-year-olds and (B) 8-year-olds. The underlying data supporting this figure can be found online in files “[Dots2Digits&Digits2Dots] _[5yo/8yo]_LogPval.nii” in “UnthresholdData” folder. (TIF) [file pbio.3001935.s006.tif]

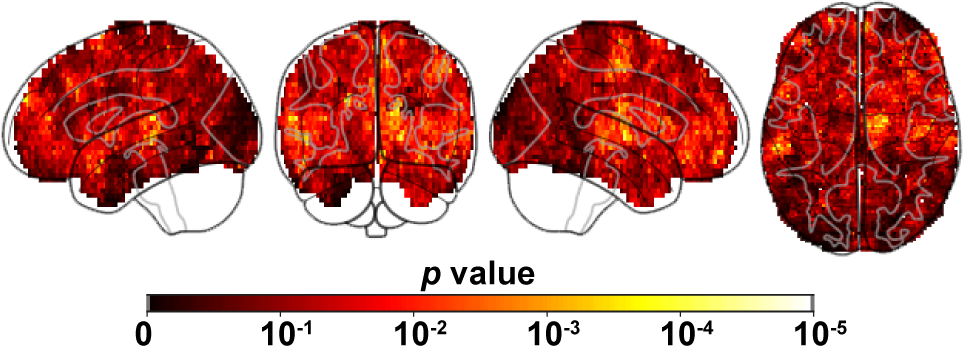

Supplement: S7 Fig — Brain regions in which activity could accurately classify between adaptation versus no-adaptation blocks of quantity in one format (dots or letters) based on training with the other format (letters or dots) (conjunction analysis), shown with unthresholded maps (for 5-year-olds). The underlying data supporting this figure can be found online in file “Dots2Letters&Letters2Dots _5yo_LogPval.nii” in “UnthresholdData” folder. (TIF) [file pbio.3001935.s007.tif]

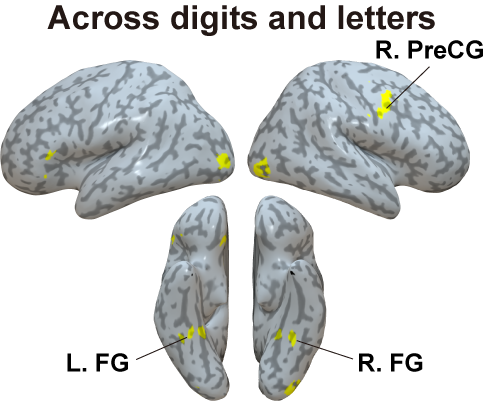

Supplement: S8 Fig — Brain regions in which activity could accurately classify between adaptation versus no-adaptation blocks of quantity in one format (letters or digits) based on training with the other format (digits or letters) (conjunction analysis). FG, fusiform gyrus; PreCG, precentral gyrus. The underlying data supporting this figure can be found online in files “RawDecAcc_LOOCV_5yo_[Letters2Digits/Digits2Letters]_[Subjects’ ID].nii” in “RawDecAcc” folder and in files “Digits2Letters&Letters2Digits_5yo_LogPval.nii” in “UnthresholdData” folder. (TIF) [file pbio.3001935.s008.tif]

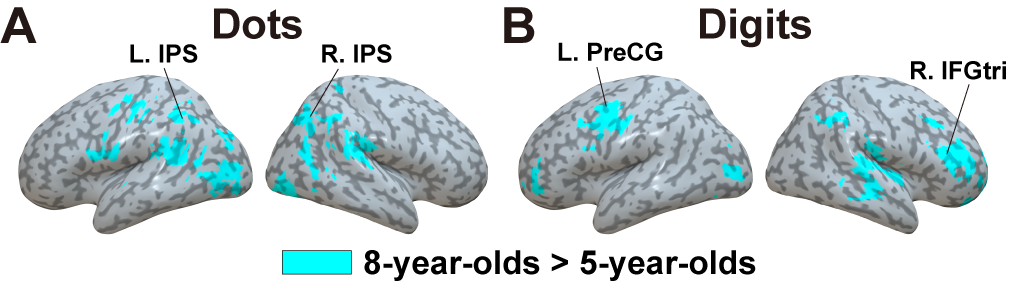

Supplement: S9 Fig — (A-B) Brain regions in which within-group decoding accuracy was larger in 8-year-olds than in 5-year-olds for (A) dots and (B) digits (based on training with the same format), after regressing out IQ and head motion parameters. (C-D) Brain regions in which activity could accurately classify between adaptation versus no-adaptation blocks of (C) dots and (D) digits in one group based on training with the same format in the other group (conjunction analysis). The underlying data supporting the panels (A-B) in this figure can be found in files “RawDecAcc_LOOCV_[5yo/8yo]_[Dots/Digits]_[Subjects’ ID].nii” in “RawDecAcc” folder and in files “Regress_[Dots/Digits]_[8yo-5yo/5yo-8yo]_LogPval.nii” in “UnthresholdData” folder. The underlying data supporting the panels (C-D) can be found online in files “RawDecAcc_[5to8yo/8to5yo]_[Dots/Digits]_[Subjects’ ID].nii” in “RawDecAcc” folder and in files “Regress_[Dots/Digits]_[5to8/8to5]_LogPval.nii” in “UnthresholdData” folder. (TIF) [file pbio.3001935.s009.tif]

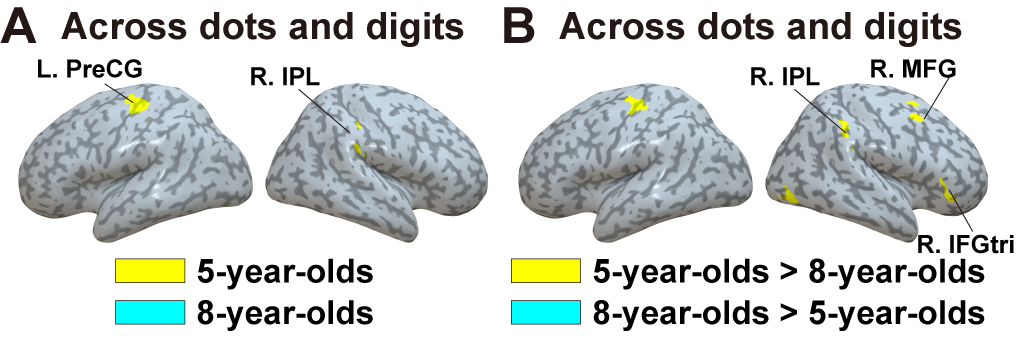

Supplement: S10 Fig — (A) Brain regions in which activity could accurately classify between adaptation versus no-adaptation blocks of quantity presented in one format (dots or digits) based on training with the other format (digits or dots) (conjunction analysis), after regressing out IQ and head motion parameters. (B) Brain regions in which between-format decoding accuracy was larger in 5-year-olds than in 8-year-olds. The underlying data supporting this figure can be found online in files “RawDecAcc_LOOCV_[5yo/8yo]_[Dots2Digits/Digits2Dots]_[Subjects’ ID].nii” in “RawDecAcc” folder and in files “Regress_Dots2Digits&Digits2Dots_[5yo/8yo/5yo-8yo/8yo-5yo]_LogPval.nii” in “UnthresholdData” folder. (TIF) [file pbio.3001935.s010.tif]

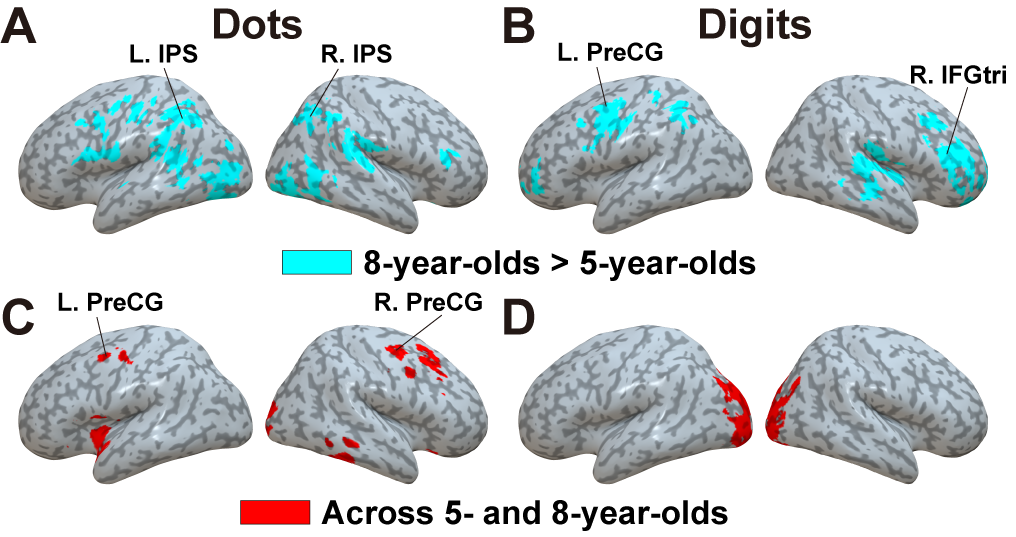

Supplement: S11 Fig — (A-B) Brain regions in which within-group decoding accuracy was larger in 8-year-olds than in 5-year-olds for (A) dots and (B) digits (based on training with the same format), using 10-fold cross-validation. Note that the between-groups decoding cannot be performed with this cross-validation method. The underlying data supporting this figure can be found online in files “RawDecAcc_10fold_[5yo/8yo]_[Dots/Digits]_[Subjects’ ID].nii” in “RawDecAcc” folder and in files “10fold_[Dots/Digits]_[5yo-8yo/8yo-5yo]_LogPval.nii” in “UnthresholdData” folder. (TIF) [file pbio.3001935.s011.tif]

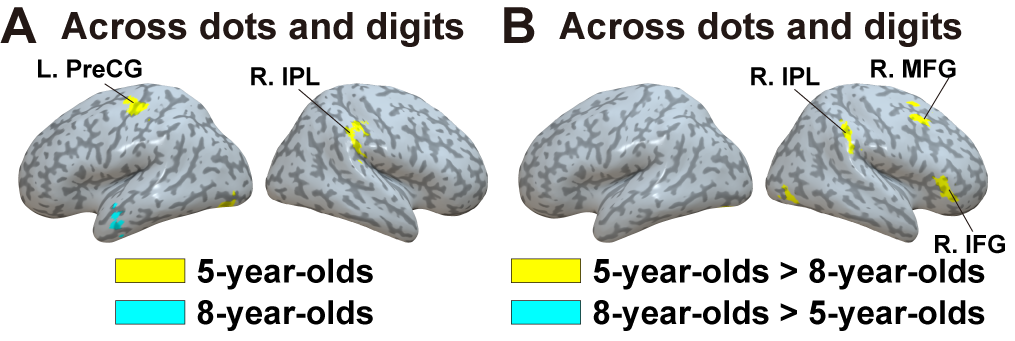

Supplement: S12 Fig — (A) Brain regions in which activity could accurately classify between adaptation versus no-adaptation blocks of quantity presented in one format (dots or digits) based on training with the other format (digits or dots) (conjunction analysis), using 10-fold cross-validation. (B) Brain regions in which between-format decoding accuracy was larger in 5-year-olds than in 8-year-olds. The underlying data supporting this figure can be found online in files “RawDecAcc_10fold_[5yo/8yo]_[Dots2Digits/Digits2Dots]_[Subjects’ ID].nii” in “RawDecAcc” folder and in files “10fold_Dots2Digits&Digits2Dots_[5yo/8yo/5yo-8yo/8yo-5yo]_LogPval.nii” in “UnthresholdData” folder. (TIF) [file pbio.3001935.s012.tif]

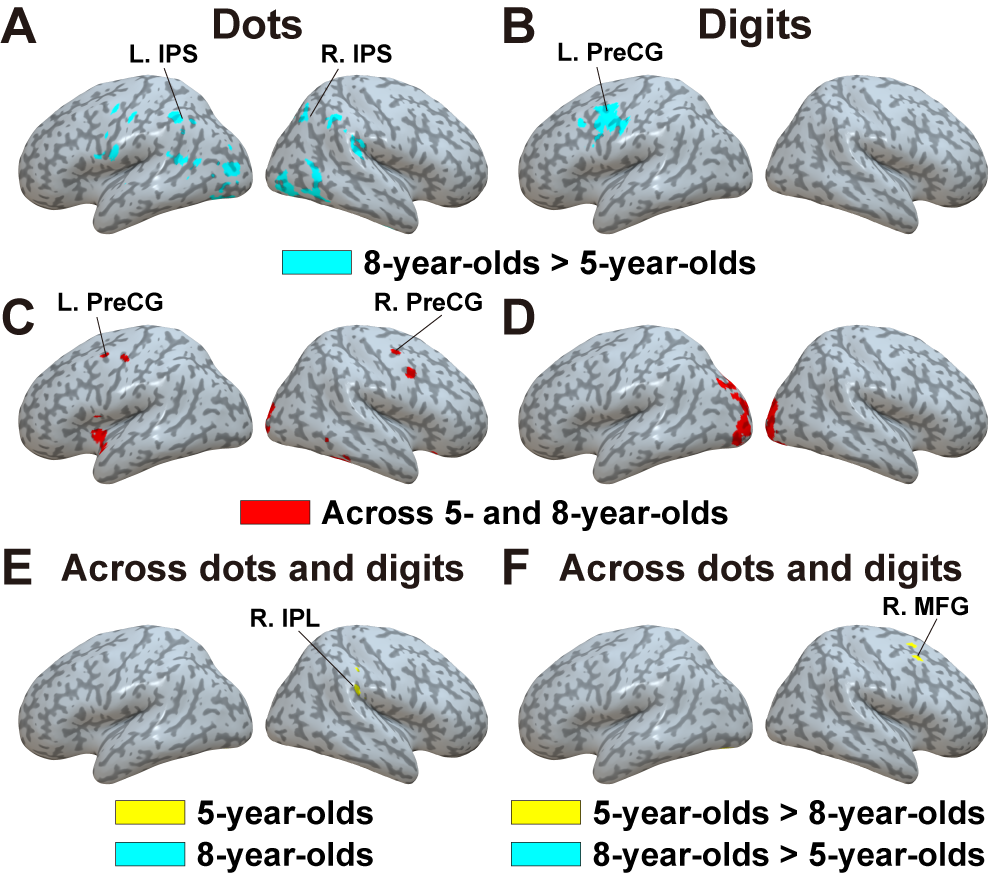

Supplement: S13 Fig — (A) Differences in within-format decoding across groups for (A) dots and (B) digits adaptation task. (C-D) Brain regions in which activity could accurately classify between adaptation versus no-adaptation blocks of (C) dots and (D) digits in one group based on training with the same format in the other group (conjunction analysis). The underlying data supporting the panels (A-B) in this figure can be found in files “RawDecAcc_LOOCV_[5yo/8yo]_[Dots/Digits]_[Subjects’ ID].nii” in “RawDecAcc” folder and in files “[Dots/Digits]_[5yo-8yo/8yo-5yo]_LogPval.nii” in “UnthresholdData” folder. The underlying data supporting the panels (C-D) can be found in files “RawDecAcc_[5to8yo/8to5yo]_[Dots/Digits]_[Subjects’ ID].nii” in “RawDecAcc” folder and in files “[Dots/Digits]_[5to8/8to5]_LogPval.nii” in “UnthresholdData” folder. The underlying data supporting the panels (E-F) can be found online in files “RawDecAcc_LOOCV_[5yo/8yo]_[Dots2Digits/Digits2Dots]_[Subjects’ ID].nii” in “RawDecAcc” folder and in files “Dots2Digits&Digits2Dots_[5yo/8yo/5yo-8yo/8yo-5yo]_LogPval.nii” in “UnthresholdData” folder. (TIF) [file pbio.3001935.s013.tif]
