# Supplementary material for: Cortical representations of numbers and nonsymbolic quantities expand and segregate in children from 5 to 8 years of age
Source: PLoS Biol. 2023 Jan 5;21(1):e3001935. doi: 10.1371/journal.pbio.3001935 (PMC9815645; doi:10.1371/journal.pbio.3001935)
Supplement: S1 Table — ACC, anterior cingulate cortex; IOG, inferior occipital gyrus; IPL, inferior parietal lobule; IPS, intraparietal sulcus; ITG, inferior temporal gyrus; L, left hemisphere; LG, lingual gyrus; MOG, middle occipital gyrus; MTG, middle temporal gyrus; PoCG, postcentral gyrus; R, right hemisphere; STG, superior temporal gyrus. (PDF) [file pbio.3001935.s014.pdf]

Anatomical Location	MNI coordinates			Peak P value (-$\log_{10}$P)	Cluster size (voxels)
	x	y	z		
R. STG	66	-26	20	4.70	137
R. ITG	66	-54	-12	4.70	183
R. IPS	58	-50	45	4.70	49
R. IOG	56	-78	-5	4.70	176
R. IPL	38	-68	45	4.70	56
R. STG	42	-12	-36	4.70	33
R. Putamen	16	12	-8	4.70	49
L. LG	-42	-88	-19	4.70	57
L. MOG	-38	-92	20	4.70	163
L. IPS	-44	-52	59	4.70	67
L. STG	-50	-44	17	4.70	37
L. MTG	-50	-56	10	4.70	36
L. PoCG	-62	2	17	4.70	71
L. ACC	-2	42	10	4.00	34
