# Supplementary material for: Cortical representations of numbers and nonsymbolic quantities expand and segregate in children from 5 to 8 years of age
Source: PLoS Biol. 2023 Jan 5;21(1):e3001935. doi: 10.1371/journal.pbio.3001935 (PMC9815645; doi:10.1371/journal.pbio.3001935)
Supplement: S2 Table — IFGtri, triangular part of inferior frontal gyrus; MFG, middle frontal gyrus; MFGorb, orbital part of middle frontal gyrus; PreCG, precentral gyrus; SFG, superior frontal gyrus; STG, superior temporal gyrus. (PDF) [file pbio.3001935.s015.pdf]

Anatomical Location	MNI coordinates			Peak P value (-$\log_{10}$P)	Cluster size (voxels)
	x	y	z		
R. STG	70	-28	10	5.67	106
R. IFGtri	58	28	31	5.67	257
R. STG	46	-20	3	5.67	82
R. MFG	46	54	13	5.67	117
R. MFGorb	32	48	-19	5.67	68
R. SFG	26	70	6	5.67	70
L. MFGorb	-38	58	-12	5.67	106
L. PreCG	-46	10	45	5.67	353
