# Supplementary material for: Cortical representations of numbers and nonsymbolic quantities expand and segregate in children from 5 to 8 years of age
Source: PLoS Biol. 2023 Jan 5;21(1):e3001935. doi: 10.1371/journal.pbio.3001935 (PMC9815645; doi:10.1371/journal.pbio.3001935)
Supplement: S3 Table — IFG, inferior temporal gyrus; MCC, middle cingulate cortex. MFG, middle frontal gyrus; MFGorb, orbital part of middle frontal gyrus; MTP, middle temporal pole; PreCG, precentral gyrus; SOG, superior occipital gyrus; SPL, superior parietal lobule. (PDF) [file pbio.3001935.s016.pdf]

Anatomical Location	MNI coordinates			Peak P value (-$\log_{10}$P)	Cluster size (voxels)
	x	y	z		
R. ITG	46	-44	-19	4.7	96
R. PreCG	40	-8	41	4.7	71
R. SOG	22	-102	13	4.7	131
L. PreCG	-38	-4	48	4.7	67
L. STG	-42	2	-12	4.7	81
R. MTP	30	16	-33	4.22	46
R. MCC	4	46	31	4.22	36
R. MFG	36	26	45	4.1	54
L. Putamen	-30	-14	6	4.1	142
R. MFGorb	14	52	-1	3.92	78
R. SPL	40	-54	59	3.8	37
R. MCC	4	-8	45	3.55	38
R. MFG	32	22	59	3.49	34
