# Supplementary material for: Cortical representations of numbers and nonsymbolic quantities expand and segregate in children from 5 to 8 years of age
Source: PLoS Biol. 2023 Jan 5;21(1):e3001935. doi: 10.1371/journal.pbio.3001935 (PMC9815645; doi:10.1371/journal.pbio.3001935)
Supplement: S4 Table — IOG, inferior occipital gyrus; LG, lingual gyrus; MOG, middle occipital gyrus; SOG, superior occipital gyrus. (PDF) [file pbio.3001935.s017.pdf]

Anatomical Location	MNI coordinates			Peak P value (-$\log_{10}$P)	Cluster size (voxels)
	x	y	z		
R. IOG	30	-96	-5	4.7	367
R. MOG	32	-84	27	4.7	
R. LG	14	-72	-5	4.7	117
L. MOG	-38	-98	-1	4.7	879
L. SOG	-24	-86	34	4.7	
