# Supplementary material for: Cortical representations of numbers and nonsymbolic quantities expand and segregate in children from 5 to 8 years of age
Source: PLoS Biol. 2023 Jan 5;21(1):e3001935. doi: 10.1371/journal.pbio.3001935 (PMC9815645; doi:10.1371/journal.pbio.3001935)
Supplement: S5 Table — IPL, inferior parietal lobule; PreCG, precentral gyrus. (PDF) [file pbio.3001935.s018.pdf]

Anatomical Location	MNI coordinates			Peak P value (-$\log_{10}$P)	Cluster size (voxels)
	x	y	z		
**5-year-olds**					
L. Cerebellum	-22	-90	-22	4.70	45
R. IPL	48	-30	31	4.40	52
L. PreCG	-40	-22	66	3.19	23
**8-year-olds**					
No significant activation					
