# Supplementary material for: Cortical representations of numbers and nonsymbolic quantities expand and segregate in children from 5 to 8 years of age
Source: PLoS Biol. 2023 Jan 5;21(1):e3001935. doi: 10.1371/journal.pbio.3001935 (PMC9815645; doi:10.1371/journal.pbio.3001935)
Supplement: S6 Table — IFGorb, orbital part of inferior frontal gyrus; IOG, inferior occipital gyrus; IPL, inferior parietal lobule; LG, lingual gyrus; MFG, middle frontal gyrus. (PDF) [file pbio.3001935.s019.pdf]

Anatomical Location	MNI coordinates			Peak P value (-$\log_{10}$P)	Cluster size (voxels)
	x	y	z		
**5-year-olds > 8-year-olds**					
R. MFG	40	10	55	4.70	88
L. LG	-24	-88	-15	4.70	23
R. IFGorb	56	32	-5	4.00	43
R. IOG	50	-86	-5	3.70	30
R. IPL	56	-38	38	3.42	24
**8-year-olds > 5-year-olds**					
No significant activation					
