# Supplementary material for: Cortical representations of numbers and nonsymbolic quantities expand and segregate in children from 5 to 8 years of age
Source: PLoS Biol. 2023 Jan 5;21(1):e3001935. doi: 10.1371/journal.pbio.3001935 (PMC9815645; doi:10.1371/journal.pbio.3001935)
Supplement: S7 Table — (PDF) [file pbio.3001935.s020.pdf]

| Anatomical Location | MNI coordinates | | | Peak P value (-$\log_{10}$P) | Cluster size (voxels) |
|---|---|---|---|---|---|
| | x | y | z | | |
| R. Thalamus | 12 | -14 | 17 | 3.92 | 39 |
