# Supplementary material for: Cortical representations of numbers and nonsymbolic quantities expand and segregate in children from 5 to 8 years of age
Source: PLoS Biol. 2023 Jan 5;21(1):e3001935. doi: 10.1371/journal.pbio.3001935 (PMC9815645; doi:10.1371/journal.pbio.3001935)
Supplement: S8 Table — FG, fusiform gyrus; IPL, inferior parietal lobule. (PDF) [file pbio.3001935.s021.pdf]

Anatomical Location	MNI coordinates			Peak P value ($-\log_{10}P$)	Cluster size (voxels)
	x	y	z		
L. FG	-26	4	-50	2.97	7
R. IPL	52	-32	34	2.94	6
