# Supplementary material for: Cortical representations of numbers and nonsymbolic quantities expand and segregate in children from 5 to 8 years of age
Source: PLoS Biol. 2023 Jan 5;21(1):e3001935. doi: 10.1371/journal.pbio.3001935 (PMC9815645; doi:10.1371/journal.pbio.3001935)
Supplement: S9 Table — FG, fusiform gyrus; IFGtri, triangular part of inferior frontal gyrus; IOG, inferior occipital gyrus; MOG, middle occipital gyrus; OC, olfactory cortex; PreCG, precentral gyrus. (PDF) [file pbio.3001935.s022.pdf]

Anatomical Location	MNI coordinates			Peak P value (-$log_{10}P$)	Cluster size (voxels)
	x	y	z		
R. FG	36	-50	-15	4.4	29
R. IOG	40	-94	-1	4.22	34
L. IFGtri	-54	20	-1	4.1	21
L. MOG	-32	-98	-1	3.92	32
R. PreCG	46	0	52	3.8	36
L. OC	-4	14	-12	3.74	18
R. PreCG	52	2	45	3.7	23
L. FG	-30	-42	-12	3.7	42
