# Supplementary material for: Cortical representations of numbers and nonsymbolic quantities expand and segregate in children from 5 to 8 years of age
Source: PLoS Biol. 2023 Jan 5;21(1):e3001935. doi: 10.1371/journal.pbio.3001935 (PMC9815645; doi:10.1371/journal.pbio.3001935)
Supplement: Attachment — Submitted filename: Predys58Decoding_PB_Response_Ver05.docx [file pbio.3001935.s023.docx]

We greatly appreciate the reviewers’ time and effort in reviewing our manuscript and providing helpful comments. Based on these comments, we have substantially revised the original manuscript. Please find our responses to the comments below, along with the related changes in the revised manuscript.

**Reviewer #1:**

**Comment 1-1:** Introduction, l.105-107: Decoding fMRI adaptation patterns using training data of 5yos and held-out independent test data of 8yos is statistically valid. But given the cross-sectional design of the present study the results reported here cannot answer the question whether neural representations of numerosity are stable (or shrink or expand) through the first four years of elementary education. My suggestion is to address this limitation to the discussion section and explicitly state that longitudinal work is needed to answer this question.

**Response 1-1:** We agree with the reviewer that longitudinal studies arguably provide the best evidence for neuro-developmental changes of activity. However, we also believe that cross-sectional designs are important as they may provide foundational evidence for differences or similarities between age groups, which can then be further explored in longitudinal designs. We note that our study remains unique in both its sample size and the fact that we examined brain activity associated with identical tasks in two groups of children from age 5 to age 8. We have addressed the reviewer’s point in three ways.

First, we made sure to acknowledge the cross-sectional nature of the design throughout the paper. This includes the title, which now reads: “Cortical representations of Arabic numerals and approximate non-symbolic quantities expand and segregate through early elementary education: A cross-sectional comparison between children aged 5 and 8”.

The cross-sectionality of the design is also reminded in the **Introduction** section (p. 3):

“In the present cross-sectional study, we used functional magnetic resonance imaging (fMRI) to investigate the emergence of the neural representations of Arabic numerals from age 5 to age 8”

It is also mentioned in the **Discussion** section (p. 15):

**“**In the present cross-sectional study, we used fMRI to examine the emergence of the neural representations of Arabic numerals through early elementary education.**”**

Second, a common concern with cross-sectional designs is that groups may differ in a variety of background measures. In response to comment 1-14, we have now provided more information regarding whether our groups differed in terms of IQ, SES, motion, and age-normalized math skills. Results are largely similar with respect to those measures (see p. 11 in the main manuscript and p.12 in the Supporting Information).

Third, we followed the reviewer’s suggestion and added the following paragraph in a new *Limitations* subsection in the **Discussion**. On p. 18, the paragraph reads:

“**Limitations**

To our knowledge, our study is unique in its investigation of the neural representations of symbolic and non-symbolic quantity using multivariate decoding in a large sample of children. However, it is also worth considering a number of potential limitations. First, although we interpret differences between 5- and 8-year-olds in terms of changes associated with learning and development, it is important to note that our design is cross-sectional. In other words, it is possible that the sample of 5-year-olds and the sample of 8-year-olds differ in a number of ways. In our view, this possibility is mitigated by the fact that we acquired a comprehensive range of background measures in each group, ensuring that they are comparable in terms of age-normalized IQ and math skills, as well as in terms of socio-economic status (SES) (see **Methods**). These measures also show that our sample was relatively diverse in terms of SES. In any case, our findings should provide the foundation for future studies investigating longitudinal changes in neural representations within the same participants.”

**Comment 1-2:** Results, Fig.2: The decoder reveals that almost the entire neocortex of 8yos contributes to classifying adaptation vs. no-adaptation dots. I do not find this neurobiologically plausible since I am not aware of literature providing evidence for visual numerosity coding in body motor and somatosensory cortex or auditory cortices. Therefore it would be highly desirable to report a statistical map showing the whole-brain voxel-wise decoding accuracy of the searchlight. Please note that this concern also applies to the other figures.

**Response 1-2:** The relatively widespread network associated with the decoding of dot patterns is indeed a striking finding from our study. However, we do not think our study is unique in finding such a pattern. In fact, a number of previous neuroimaging studies have shown similar distributed decodability across the whole brain, including around the central sulcus and temporal cortex. For example, using a searchlight decoding approach, Bulthé et al. (NeuroImage, 2014) reported accurate decodability of non-symbolic quantity across the frontal, parietal, temporal, and occipital cortices (see Fig. 3). Harvey and Dumoulin (Nat Human Behav, 2017) also reported topographic numerosity maps not only in the parietal cortex but also in the frontal and temporal cortices. Kovas et al. (PLOS ONE, 2009) and Kersey and Cantlon (JNS, 2017) found that a distributed system is associated with the processing of non-symbolic numerosity in children from age 3 to age 10, including the parietal, occipital, temporal, and frontal cortices. Therefore, our findings are consistent with these previous reports. It is important to note that a common feature of these studies (as well as ours) is that they all involve *whole-brain* univariate or multivariate analyses. This contrasts with several studies that have explicitly focused on a subset of regions of interest (e.g., within the occipital or parietal cortex; Knops, Science 2009; Castaldi et al., eLife 2019) or have not measured fMRI signal across the whole brain (Eger et al., Curr Biol 2009). These studies may thus have missed representations of non-symbolic quantities in a number of regions outside the parietal and occipital cortex (including the temporal and frontal cortex).

To elaborate of this point, we added the following paragraph in the **Cortical representations of symbolic and non-symbolic quantity expand from age 5 to age 8** subsection of the Discussion (pp. 15-16):

“On the one hand, we found that Arabic numerals and non-symbolic quantities were represented in the bilateral occipital and parietal cortices in both 5- and 8-years-olds. These areas have long been the focus of previous studies that investigated neural activity associated with symbolic and non-symbolic quantity [[6,9,13–16]](https://paperpile.com/c/lxwFL4/377Vj+qco1p+5oR6B+SvgiR+5TzKK+tHJoZ). However, our results suggest the involvement of a more distributed brain system that also includes bilateral regions of the temporal and frontal cortices. It is interesting to note that our study is not the first to show that the temporal and frontal cortices are involved in the processing of symbolic and non-symbolic quantity, notably in children. For example, both Kovas et al. [[32]](https://paperpile.com/c/lxwFL4/QGxw) and Kersey and Cantlon [[4]](https://paperpile.com/c/lxwFL4/q92RH) found a distributed system associated with the processing of non-symbolic numerosity in children from age 3 to age 10, including the parietal, occipital, temporal, and frontal cortices. Bulthé et al. [[12]](https://paperpile.com/c/lxwFL4/VCBhC), though focusing on adults, also reported that non-symbolic quantity could be decoded across the whole brain. Importantly, we found some degree of stability in the neural representation of non-symbolic quantity. Specifically, decoders trained with brain activity from 5-year-olds accurately classified between activity associated with adaptation versus no-adaptation blocks of dots in 8-year-olds, and vice-versa. This was notably the case in the right SPL and bilateral PreCG. However, no representational stability was found for the neural representation of Arabic numerals in the parietal, frontal, or temporal cortex between age 5 and age 8, most likely because symbolic numerical skills change massively during that time.”

Although the whole-brain statistical maps of between-format decoding analysis were already provided in Supporting Information (Figures S3 and S4), we have now added unthresholded statistical maps of within-format analysis as Figures S1 and S2, and across-group analysis as Figure S4.

We also added the following sentences in the **Within-format searchlight decoding** subsection of the results (p. 7, 8):

**“Figures S1** and **S2** show the unthresholded within-format accuracy maps for 5-year-olds (Dots, **Figure S1A**; Digits, **Figure S1B**) and 8-year-olds (Dots, **Figure S2A**; Digits, **Figure S2B**).”

“**Figure S4** shows the unthresholded across-group accuracy maps for dots (**Figure S4A**) and digits (**Figure S4B**).”

Finally, we have added the following citation:

“[Kovas Y, Giampietro V, Viding E, Ng V, Brammer M, Barker GJ, et al. Brain correlates of non-symbolic numerosity estimation in low and high mathematical ability children. PLoS One. 2009;4: e4587. doi:](http://paperpile.com/b/lxwFL4/QGxw)[10.1371/journal.pone.0004587](http://dx.doi.org/10.1371/journal.pone.0004587)”

**Comment 1-3:** Methods, l.567-572: In cluster-based multiple comparison correction an uncorrected cluster-forming voxel-wise threshold of P < 0.005 is considered as too liberal to ensure robust replication (Eklund et al. 2016 PNAS). Please provide a reference proving that this problem does not arise in permutation-based multiple comparison correction or apply conservative voxel-based thresholding (family-wise error or false discovery rate).

**Response 1-3:** We thank the reviewer for bringing up this important point, and we apologize for not being sufficiently clear in the original manuscript. In fact, the study by Eklund et al. (2016) does provide support for the use of permutation-based multiple comparison correction instead of parametric testing. Indeed, Eklund et al. (2016) did not show that an uncorrected voxel-wise threshold of P < 0.005 was too liberal for controlling for multiple comparisons *in general*. They showed that the issue regarding a liberal cluster-based thresholding specifically arises with parametric testing. As shown in the Figure 1 in Eklund et al. (2016), parametric methods are much more sensitive to cluster-defining threshold (i.e., voxel-wise threshold) than permutation tests. As stated by Eklund et al. (2016, p. 7900), “by comparison, the nonparametric permutation test is found to produce nominal results for both voxelwise and clusterwise inference for two-sample t tests, and nearly nominal results for one-sample t tests.” According to Eklund et al. (2016), the issue with parametric testing is caused by the assumption of constant spatial smoothness across the brain (which is not the case for non-parametric testing). Therefore, Eklund et al. (2016, PNAS) indicate that our permutation-based multiple comparison correction is valid and much more robust to variations in cluster-forming thresholds than parametric methods (which is why we used the method).

To clarify the above point, we added the following text in the **Searchlight decoding analysis** subsection from the Methods (p. 25):

“Statistical significance was assessed using cluster-wise inference via a non-parametric sign permutation test [[57,58]](https://paperpile.com/c/lxwFL4/EvBfh+XtYMf). Unlike parametric tests, which assume constant spatial smoothness across the brain, non-parametric tests do not make that assumption and are therefore much less sensitive to the cluster-defining threshold [[59]](https://paperpile.com/c/lxwFL4/gfti).”

We also added the following citation:

“Eklund A, Nichols TE, Knutsson H. Cluster failure: Why fMRI inferences for spatial extent have inflated false-positive rates. Proc Natl Acad Sci U S A. 2016;113: 7900–7905. doi:10.1073/pnas.1602413113”

**Comment 1-4:** Results, general: I consider it as a problem that it is not reported how well the participants can identify and discriminate dots and digits at the behavioral level. I find it unrealistic to expect that age is perfectly correlated with numerosity detection performance given that there are pronounced individual differences in math ability. My recommendation is to address this limitation in the discussion section. I would state that the results reported here do not allow to draw conclusions about numerosity detection ability.

**Response 1-4:** We agree with the reviewer that, although both symbolic and non-symbolic numerical skills increase from age 5 to age 8 (Halberda & Feigenson, 2008, Dev. Psych.; Geary et al. 2012, J. Educ. Psychol.), age is not perfectly correlated with performance. We do not think we have made such a claim in the original manuscript and apologize if any of our writing might have suggested otherwise. In fact, we deliberately chose tasks in which behavioral performance was absent to avoid confounding activity with differences in performance between 5- and 8-year-olds. The goal of our manuscript was to evaluate the *neural* relation between symbolic and non-symbolic quantity processing and how it may change in young children.

Following the reviewer’s suggestion, we have now included a paragraph in the **Limitations** subsection of the Discussion to acknowledge the reviewer’s point (p. 19):

“Third, although passive adaptation tasks are well suited to examine differences in brain activity between children of different ages, a drawback is that they do not make it possible to collect indicators of task performance. Therefore, our results do not allow us to make any conclusion regarding how well the participants could identify and discriminate symbolic and non-symbolic quantities at the behavioral level. However, there is overwhelming evidence that performance on symbolic and non-symbolic quantity tasks increases between the ages of 5 and 8 [[42,43]](https://paperpile.com/c/lxwFL4/XwgH+PcUl), which is also suggested by the standardized measures collected outside of the scanner in the present study. Of course, this does not mean that there are no individual differences in performance within each age group. Even though assessing the relation between those individual differences and decoding performance would be interesting, such analyses would require much larger sample sizes than those used in the present investigation [[44]](https://paperpile.com/c/lxwFL4/XwOQ).”

Although the nature of our tasks does not make it possible to assess in-scanner performance, we did collect behavioral data on children’s symbolic and non-symbolic numerical skills outside of the scanner (as part of the larger project the study comes from). Specifically, children’s numerical ability was estimated using a subset of the TEDI-MATH (Noël and Grégoire 2015) in 5-year-olds and of the ZAREKI-R in 8-year-olds (Von Aster 2005). Results show that both groups have average performance compared to their expected age. We have now included these data in the **Results** section in the new manuscript to help characterize our samples (p. 6):

“Measures of mathematical skills included standardized assessments of symbolic and non-symbolic numerical competence from two age-appropriate batteries, the TEDI-MATH for 5-year-olds [[27]](https://paperpile.com/c/lxwFL4/fFfk) and the ZAREKI-R for 8-year-olds [[28]](https://paperpile.com/c/lxwFL4/fewY) (see **Methods**). For 5-year-olds, measures involved symbolic number comparison and non-symbolic quantity estimation. For 8-year-olds, measures involved symbolic number comparison and non-symbolic quantity comparison. For non-symbolic competence, the average standardized score was 104 in 5-year-olds (SD = 5, range = 85–105) and 108 in 8-year-olds (SD = 18, range = 57–123). For symbolic competence, the average standardized score was 102 in 5-year-olds (SD = 13, range = 57–109) and 107 in 8-year-olds (SD = 7, range = 83–111). Therefore, although scores cannot be directly compared between groups due to the use of different subtests and batteries, average scores of 5- and 8-year-olds were in line with typical age expectation. Note that the equivalent standardized scores also indicate that raw performance was higher for 8- than 5-year-old.”

Finally, tests are described in the **Methods** section (p. 21):

“Symbolic and non-symbolic numerical skills were estimated using subsets of TEDI-MATH [[27]](https://paperpile.com/c/lxwFL4/fFfk) for 5-year-olds and subtests of the ZAREKI-R for 8-year-olds [[28]](https://paperpile.com/c/lxwFL4/fewY). TEDI-MATH subtests included the “Estimation visuelle de quantites” and the “Comparaison de deux nombres écrits". The former subtest requires children to estimate a number of objects presented visually within two or five seconds, while the latter requires children to decide which of two written symbolic numbers is the largest. ZAREKI-R subtests included the “Comparaison de patterns de points dispersés” and the “Comparaison de nombres arabes”. The former subtest requires children to decide which of two dot patterns included the largest number of dots within one second, while the latter requires children to decide which of two written symbolic numbers is the largest.”

We added the following citations:

“Noël M-P, Grégoire J. TediMath Grands, Test diagnostique des compétences de base en mathématiques du CE2 à la 5e. dial.uclouvain.be; 2015. Available: https://dial.uclouvain.be/pr/boreal/object/boreal:167790”

“Von Aster M. ZAREKI-R: Batterie pour l’évaluation du traitement des nombres et du calcul chez l'enfant. ECPA, les Éditions du centre de psychologie appliquée; 2005.”

“[Halberda, Feigenson. Developmental change in the acuity of the“ Number Sense”: The Approximate Number System in 3-, 4-, 5-, and 6-year-olds and adults. Dev Psychol. 2008. Available:](http://paperpile.com/b/lxwFL4/XwgH) <https://psycnet.apa.org/record/2008-12114-023>”

“[Brankaer C, Ghesquière P, De Smedt B. Symbolic magnitude processing in elementary school children: A group administered paper-and-pencil measure (SYMP Test). Behav Res Methods. 2017;49: 1361–1373. doi:](http://paperpile.com/b/lxwFL4/PcUl)[10.3758/s13428-016-0792-3](http://dx.doi.org/10.3758/s13428-016-0792-3)”

“[Marek S, Tervo-Clemmens B, Calabro FJ, Montez DF, Kay BP, Hatoum AS, et al. Reproducible brain-wide association studies require thousands of individuals. Nature. 2022;603: 654–660. doi:](http://paperpile.com/b/lxwFL4/XwOQ)[10.1038/s41586-022-04492-9](http://dx.doi.org/10.1038/s41586-022-04492-9)”

**Comment 1-5:** Results, general: In my view, there is a mismatch between the surface renderings of the classification accuracy maps and the neuroanatomical labels assigned by the authors. For example, in Fig. 3B, L.DLPFC seems to be the premotor cortex instead. Please provide MNI coordinates and use an atlas to assign neuroanatomical labels.

**Response 1-5:** We thank the reviewer for bringing up this issue and acknowledge a localization inaccuracy here. Based on the automated anatomical labeling (AAL) atlas, the DLPFC in Table S1 and S5 should have been labeled as the precentral gyrus (PreCG). We have corrected this on the Tables and Figures and have carefully checked all anatomical localization based on the AAL atlas. Notably, since the DLPFC is a functionally defined label (Cieslik et al., Cereb Cortex 2013), we now refer to the anatomical label of the MFG and PreCG instead. We replaced “DLPFC” in Figure 3 with “PreCG,” “DLPFC” in Figure 4 with “PreCG” (Figure 4A), and “MFG” (Figure 4B). The anatomical labels of “IFG” in Figures 3 and 4 were replaced by “IFGtri.” The anatomical label “IPS” in Figure 3 was replaced by “SPL.” Figures in Supporting Information were also replaced in the same manner. Descriptions of anatomical labels in the main manuscript were also changed accordingly. Although we already provided MNI coordinates of between-format decoding maps in the original manuscript, we now also provide MNI coordinates of within-format decoding maps in the new manuscript (Tables S1-S4 in the Supporting Information).

We further modified the following text in the **Within-format searchlight decoding** subsection of the results (p. 8):

“(**Figures 3A, B, Table S1, S2**)”

“(**Figures 3C, D, Table S3, S4**; see **Figure S3** for accuracy maps associated with each direction-specific analysis)”

Regarding the anatomical labeling, we added the following text in (p. 25)

“Anatomical labels were determined based on the automated anatomical labeling (AAL) atlas [62].”

We added the following citation:

“[Tzourio-Mazoyer N, Landeau B, Papathanassiou D, Crivello F, Etard O, Delcroix N, et al. Automated anatomical labeling of activations in SPM using a macroscopic anatomical parcellation of the MNI MRI single-subject brain. Neuroimage. 2002;15: 273–289. doi:](http://paperpile.com/b/lxwFL4/SlkR)[10.1006/nimg.2001.0978](http://dx.doi.org/10.1006/nimg.2001.0978)”

**Comment 1-6:** Results, Fig.4: I think that the authors cannot infer a group difference from a significant finding in one group (5yo) and a null finding in the other group (8yo). To make this claim they would have to report a statistical index showing that the decoding accuracy in one group is significantly higher. Is this the case?

**Response 1-6:** We once again agree with the reviewer. In fact, we did report a direct group comparison in Figure 4B in the original manuscript (using a two-sample permutation test). We apologize if that was not clear enough and have attempted to make this clearer in the revised manuscript. Specifically, we found that the 5yo > 8yo contrast showed a significant difference in between-format decoding accuracy in several brain regions, including the right IPL. In other words, there was a significant group difference (we did not simply infer a group difference from a significant finding in one group and a null finding in the other group).

This result is described in the **Between-format searchlight decoding** subsection of the Results (p. 10):

“A direct comparison of accuracy maps between groups showed higher between-format decoding accuracy for 5-year-olds than for 8-year-olds in the right IPL, as well as in regions of the right MFG and the triangular part of inferior frontal gyrus (IFGtri) (**Figure 4B** and **Table S6**). No region showed higher between-format decoding accuracy for 8-year-olds than for 5-year-olds.”

The finding is also discussed on p. 16, where we now make it clearer that we infer a group difference from a direct comparison:

“Specifically, although we found a region of the right IPL in which quantity was represented in a format-independent manner in 5-year-olds, this format-independence was absent in 8-year-olds. Between-format decoding accuracy was also significantly higher in 5-year-olds than in 8-year-olds, indicating that the groups statistically differed when compared with each other’s.”

**Comment 1-7:** Results, l.254-267: While attention did not differ within each age group, I think that the authors cannot rule out that the different fMRI patterns might not be explained by between-group differences in numerosity detection, but by between-group differences in attention. Moreover, the fronto-parietal cortices house several other systems (incl. working memory and metacognition (theory of mind)) so that differences in these domains are plausible alternative explanations that are not controlled here. I consider it as very important to acknowledge this substantial limitation in the discussion. Also, although IQ differences between the age groups did not reach significance it would nevertheless be desirable to include Iq scores as covariates of no interest in the models to control for an effect of general cognitive ability.

**Response 1-7:** We acknowledge that between-group differences in neural representations might be affected by a number of factors. As the reviewer, we were concerned about attentional levels, which is why we embedded a target detection task in the design of the study. We believe that such a task is probably the best indicator of attentional levels we can have within an otherwise passive adaptation task. We showed that (1) target detection rates of the three tasks were largely above chance for both 5- and 8-year-olds (Wilcoxon signed-rank test, Bonferroni correction for multiple comparisons, adjusted *P* < 0.001), and that (2) there was no between-task difference in target detection rates. Therefore, there is no indication from behavioral data that either group did not pay attention to the task. We have now added the sentence below in the **Control analyses** subsection of the Results (p. 12):

“Target detection rates of three tasks were largely above chance in each group (Wilcoxon signed-rank test, Bonferroni correction for multiple comparisons, adjusted *P* < 0.001)”

We also note that two results are inconsistent with the idea that attentional differences explain our differences in between-format decoding between 5- and 8-year-olds. First, if attention was driving the between-group difference in between-format decoding, such a finding would have been more likely in 8-year-olds than in 5-year-olds (as levels of attention are expected to increase with age). Yet, we found larger between-format decoding in 5-year-olds than in 8-year-olds.

Second, the between-format decoding analysis of dots and letters (Control Analysis, Figure 5) is also informative regarding the question of attentional differences between groups and whether they caused our results. If the decoding accuracy in the right IPL was caused by differences in attentional levels (or by the other general cognitive factors such as metacognition or working memory), such effect would also be found in the other non-numerical modality (i.e., letter). Yet, as shown in Figure 5B, we found higher decodability between dots and digits than between dots and letters in the right IPL. Therefore, our results in the right IPL are difficult to explain by a difference in attentional levels.

We added the following paragraph in the **Limitations** subsection of the Discussion (pp. 18-19):

“Second, studying task-related changes in neural processing across development is challenging because such changes are often confounded with differences in behavioral performance [[20]](https://paperpile.com/c/lxwFL4/USOgi). For this reason, we used adaptation tasks that do not require any overt behavior. As a result, we interpret differences between age groups as coming from differences in the passive processing of symbolic and non-symbolic quantity. However, it could be argued that such differences might also be (at least partly) driven by differences in attentional levels between groups. We think that this is unlikely because our analysis of the concurrent target detection task showed that even 5-year-olds were paying significant attention to the stimuli. It is also interesting to note that if attention was driving the between-group differences in between-format decoding, such a finding would have been more likely in 8-year-olds than in 5-year-olds (as levels of attention are expected to increase with age). Yet, we found larger between-format decoding in 5-year-olds than in 8-year-olds. Nonetheless, we acknowledge that even passively processing symbolic and non-symbolic quantity might involve a number of cognitive processes that are inherent to the processing of these stimuli (e.g., working-memory) and are therefore difficult (if not impossible) to control for.”

We also modified the following paragraph in the **Overlap in the cortical representations of symbolic and non-symbolic quantity in young children is specific to numerical information** subsection of the Discussion (p. 17):

“Unlike most previous fMRI adaptation studies [[3,9,31]](https://paperpile.com/c/lxwFL4/jUvHT+qco1p+Udmdr), our study focuses on multivariate activity patterns during adaptation periods rather than univariate activity associated with a deviant stimulus. Although this makes our design particularly robust and well suited to pediatric neuroimaging, a downside is that we could not evaluate whether decoding was influenced by numerical properties of the stimuli (e.g., distance between quantities). Therefore, it could be argued that the overlap between the neural representations of dots and digits in 5-year-olds is mainly due to domain-general adaptation effects. To rule out this possibility and assess the specificity of the overlap between symbolic and non-symbolic quantity, we also presented participants with symbolic stimuli that did not involve any quantitative information, i.e., letters. Critically, we did not find any significant between-format decoding between dots and letters anywhere in the brain (and we found higher decoding accuracy between dots and digits than that between dots and letters in the right IPL). This indicated that the overlap between the neural representations of dots and digits in the IPL of 5-year-olds was specific to symbolic numbers (**Figures 4, 5**).”

Based on the reviewer’s suggestion, we also performed an additional analysis, including IQ and head motion parameters as covariates of no-interest (**Figures S11, S12**). This analysis replicated our main results. Notably, we found significant between-age decoding accuracy in the bilateral PreCG and higher between-format decoding accuracy for 5-year-olds in the right IPL

We added the following text in the **Control analyses** subsection (p. 13):

“Finally, although 5- and 8-year-olds did not differ with respect to either in-scanner motion (see **Methods**) or standardized IQ (see above), it remains possible that differences between groups in terms of motion or general cognitive functioning (i.e., IQ) might have affected our results. To exclude these possibilities, we performed a multiple linear regression analysis (with intercept) using the six head motion parameters and IQ as independent variables, as well as the decoding accuracy of each participant as a dependent variable. The predicted value associated with the independent variables was subtracted from the original decoding accuracy and was used as a new decoding accuracy. The group-level statistical significance was evaluated in the same manner as described above. Again, this analysis revealed very similar results, with significant between-age decoding accuracy in the bilateral PreCG and higher between-format decoding accuracy for 5-year-olds in the right IPL (**Figures S11, S12**). Therefore, differences between children’s in-scanner motion or IQ did not appear to have influenced our results.”

**Comment 1-8:** Methods, Adaptation task: I find it problematic that the number of dots (6-60) did not match the numerosities corresponding to the digits (1-8). I fear that these differences wrt the numerosities could explain the lack of format independence in the older children. Please discuss this in extenso in the discussion.

**Response 1-8:** The reviewer is correct that symbolic and non-symbolic stimuli are not similar with respect to the absolute quantity they represent. In fact, this aspect of our design is an unavoidable feature of comparing approximate non-symbolic quantity processing and symbolic quantity processing in young children. The goal of the paper was to test the relation between the “approximate number system” (ANS) and the neural representations of symbolic numbers. By definition, the ANS can only be tested with quantities that have to be approximated. That is, they must be large enough to not be either subitized or counted, which would surely be the case with quantities between 1 and 8 (Nunez, Trends Cogn Sci 2017). Note that this is why many numerical cognition studies have used large numerosities in general when investigating the ANS (e.g., Kersey, JNS 2017). It was not feasible to match these quantities with symbolic numbers in children as young 5, because most of them have not learned double-digit numbers yet. In other words, there was no other way to test symbolic numerical processing in children that young than using Arabic numerals. Note that our analyses decoded differences between adaptation and non-adaptation conditions within each task. In other words, the different quantities represented by digits and dots stimuli were never directly compared. We have addressed the reviewer’s comment in a several ways in the revised manuscript.

First, we have emphasized throughout the manuscript that the goal of the current study is to investigate the *approximate* number system. For example, the title now reads:

“Cortical representations of Arabic numerals and approximate non-symbolic quantities expand and segregate through early elementary education: A cross-sectional comparison between children aged 5 and 8”

The fact that we focus on approximate non-symbolic quantities is also emphasized throughout the paper. For instance, in the introduction (p. 3):

“We also aimed to assess the relation between the representations of Arabic numerals and approximate non-symbolic quantity during that period.”

Second, to clarify the choice of stimuli, we added the following text in the **Adaptation task** subsection of the Methods (pp. 21-22):

“That is, set sizes in the dot adaptation task typically exceeded the subitizing range and the counting range [17]”

“In the digit adaptation task, stimuli were digits ranging from 1 to 8. Because these are always taught in French pre-K and K, they are known to children as young as 5 (which is not necessarily the case for double-digit numbers).”

Third, we added the following paragraph in the **Limitations** subsection of the Discussion (p. 19) to acknowledge the reviewer’ comment:

“Finally, the main goal of the present study was to test the influential hypothesis that learning symbolic numbers builds on an approximate sense of quantity that is associated with the ANS [[5]](https://paperpile.com/c/lxwFL4/ECnbf). To elicit ANS processing, we chose non-symbolic stimuli based on previous studies in which participants are typically presented with relatively large sets of dots that cannot be enumerated (thereby preventing exact symbolic processing). However, because young children in kindergarten do not master the symbolic place-value notation, our symbolic stimuli did not include double-digit numbers and were restricted to Arabic numerals. In other words, symbolic and non-symbolic stimuli did not represent the same quantities. Therefore, although our results can inform about the relation between the neural representations of Arabic numerals and approximate non-symbolic quantities in young children, our conclusions might not extend to the relation between exact symbolic and exact non-symbolic quantities.”

**Comment 1-9:** Methods, fMRI data preprocessing: Surprisingly, more 8yos than 5yos were excluded due to head motion and head motion was still higher in the remaining 8yos. Please include head motion indices as covariates of no interest in all models.

**Response 1-9:** There was no significant difference between 5- and 8-year-old children in terms of head motion (Wilcoxon rank-sum test, Bonferroni correction for multiple comparisons, adjusted *p* = 0.25, 1.00, 0.25 for x, y, z translations, respectively; adjusted *p* = 0.40, 0.10, 0.40 for pitch, yaw, and roll rotations, respectively). We have now included this comparison in the revised version of the manuscript. In the **fMRI data preprocessing** subsection of the Methods (p. 23), the new sentence reads:

“There was no significant difference in head motion between 5- and 8-year-old children (Wilcoxon rank-sum test, Bonferroni correction for multiple comparisons, adjusted *p* = 0.25, 1.00, 0.25 for x, y, z translations, respectively; adjusted *p* = 0.40, 0.10, 0.40 for pitch, yaw, and roll rotations, respectively).”

We also found that there was a mistake in the previous version of the paper and thank the reviewer for pointing it out to us: there was in fact more 5-year-olds excluded than 8-year-olds (and not the other way around). We modified the following text in the **fMRI data preprocessing** subsection (p. 23):

“Participants with outliers in more than 20% of volumes were excluded from the analyses (5-year-olds, n = 21; 8-year-olds, n = 2)”

Finally, we also performed an additional analysis in which we included the six head motion parameters as the covariates of no-interest (see new **Figures S11, S12**). We obtained similar results, as described in response to **Comment 1-7**.

**Comment 1-10:** General remark: The manuscript would benefit from proofreading by a native English speaker. There are a number of semantic and grammatical mistakes as well as stylistic flaws in the text.

**Response 1-10:** We asked a native English speaker to proofread our new manuscript.

**Comment 1-11:** Results, general: Are the results robust to the cross validation procedure chosen here? Please provide the results obtained using 10-fold cross validation (Knops et al. 2009).

**Response 1-11:** Based on the reviewer’s suggestion, we performed additional analyses using 10-fold cross-validation and found similar results. We included the results of the 10-fold cross-validation in Figures S9 and S10 in the Supporting Information.

We also added the following text in **Control analyses** subsection of the Results (p. 12):

“Third, it is possible that decoding accuracy might depend (to some extent) on the type of cross-validation procedure. To evaluate whether this was the case, we performed a 10-fold cross-validation procedure [[29]](https://paperpile.com/c/lxwFL4/9KV0) in addition to the main LOOCV procedure. We obtained very similar results (**Figures S9, S10**). Notably, we replicated our main finding of significantly higher between-format decoding accuracy in the right IPL for 5-year-olds, but not 8-year-olds.”

We added the following citation:

“[Knops A, Thirion B, Hubbard EM, Michel V, Dehaene S. Recruitment of an area involved in eye movements during mental arithmetic. Science. 2009;324: 1583–1585. doi:](http://paperpile.com/b/lxwFL4/9KV0)[10.1126/science.1171599](http://dx.doi.org/10.1126/science.1171599)”

**Comment 1-12:** Discussion, l.322-325: I do not understand why larger numerosity responses in 8yo vs 5yo are consistent with the idea that the acquisition of symbolic number skills refine the cognitive mechanisms supporting non-symbolic quantity processing. Please clarify.

**Response 1-12:** We apologize for the lack of clarity. Our data show an expansion of the cortical representations of both symbolic and non-symbolic quantities in 8-year-olds compared to 5-year-olds (**Figure 2**). Although the expansion of symbolic representations is expected because older children have more experience with symbolic numbers, the expansion of non-symbolic representations may be more surprising because such representations are largely thought to come from an evolutionarily older system that some may believe develop before formal education.

However, the paper cited in this paragraph (Piazza et al., Psychol Sci 2013) suggests that the non-symbolic system can also be sharpened with formal education. Indeed, Piazza et al. (Psychol Sci, 2013) measured numerosity estimation acuity in children and adult members of an Amazonian indigene group (the Mundurucu). Mundurucu participants with longer formal education (i.e., experience in symbolic numbers), regardless of their age, showed a more precise numerical estimation acuity. According to the authors, this result suggests that “education plays a significant role in sharpening the sense of approximate numerical quantity” (Piazza et al., Psychol Sci 2013, p. 6). Although our findings are about neural representations and not behavioral performance, we find the argument relevant to discuss our current results.

To clarify the relation between the current results and Piazza et al. (Psychol Sci 2013), we added the following text in the **Discussion** section (p. 16):

“Note, however, that there was also an expansion of the cortical regions representing non-symbolic quantity, most notably around the IPS. Although non-symbolic numerosity representations are evolutionarily old and therefore emerge early in children [[1–4]](https://paperpile.com/c/lxwFL4/NGm7G+Vactk+jUvHT+q92RH), this finding is consistent with the idea that the acquisition of symbolic number skills during the first four years of education may still refine the cognitive mechanisms supporting non-symbolic quantity processing [[34]](https://paperpile.com/c/lxwFL4/nNAUl)”

**Comment 1-13:** Discussion, l.378-379: I do not understand why the fact that the authors observed between-format decoding in both hemispheres can be explained by the fact that orthographic features are shared by both digits and letters. Please explain.

**Response 1-13:** We apologize to the reviewer for the lack of clarity. This speculation was not central to our argument and have now removed it from the discussion.

We modified the following text in **Cortical representations of digits and letters overlap in young children** subsection in the Discussion (pp. 17-18):

“Interestingly, the lack of overlap between representations of dots and letters was not due to a failure to find shared representations between letters and any other types of stimuli. Indeed, in 5-year-olds, there was significant between-format decoding across letters and digits in both the bilateral FG and the right PreCG (**Figure S8**). This suggests that both of these regions might be involved in representing symbolic information more generally. Consistent with this idea, several studies notably suggest that the FG might include areas dedicated to the identification of both words and numerals from low-level visual features [[38–41]](https://paperpile.com/c/lxwFL4/ZisUG+iONp4+O8wMZ+BFj1n). It is also possible that the neural overlap between digits and letters in the right PreCG is due to a common representation of stimuli features, independently of quantity (e.g., visual features).”

**Comment 1-14:** Methods, Participants: Were the 49 children that dropped out during fMRI comparable to the 89 children that did not drop out in terms of their cognitive ability and socioeconomic background?

**Response 1-14:** Among the 49 children who were excluded, 39 were 5-year-olds and 10 were 8 years old. We compared the full-scale IQ between children who were included and children who were excluded from MRI testing. There was no significant difference between these groups (Wilcoxon rank-sum test, *P* = 0.06). Regarding socioeconomic status (SES), we measured parents’ income. Again, there was no significant difference in parental income between children who were included and children who were excluded from MRI testing (*P* = 0.69).

To describe the SES result, we added the following text in the **Participants** subsection of the Methods (p. 21):

“Family SES of children was assessed using the income of the parent who was with the child during testing. Parental income ranged from 500 to 5,500 euros (mean = 1,893, SD = 1,164) across all families included in the main analyses. Given that the median monthly income in France is about 1,700 euros [[44]](https://paperpile.com/c/lxwFL4/dpwr), family SES ranged from low to high. There was no difference between children who were included in the final analyses and those who were excluded with respect to parental income (Wilcoxon rank-sum test, *p* < 0.69).”

We added the following citation:

“[Robin M. More people live in a median household in the western regions. Insee Focus. 2019.](http://paperpile.com/b/lxwFL4/dpwr)”

**Comment 1-15:** Methods, Behavioral testing: It should be mentioned that the children enrolled in the present study had above average intelligence. Could this be explained by a selection bias?

**Response 1-15:** We thank the reviewer for this remark. Although the reviewer is correct that the children included in the study had an average IQ slightly above the norm (5-year-olds: 109, 8-year-olds: 112), it is important to keep on mind that (1) groups were comparable with respect to their average IQ (Wilcoxon rank-sum test, *p* = 0.18) and (2) there was still a range of IQs within each group (5-year-olds, range = 76–146; 8-year-olds, range = 83–135). We recruited the children and their parents through flyers sent to schools and advertisements on social media and did not have an explicit criterion regarding their intelligence or cognitive ability. However, we acknowledge that there might always be a possible selection bias, such that families interested in participating in our experiments might generally have more motivation for science compared to the average population. Critically, however, the fact that our results remain unchanged when IQ is included as a covariate suggests that IQ might not have a large influence on our results (see Response 1-7)

We have added the following sentence in the **Participants** subsection of the Methods (p. 20) to five information about the recruitment process:

“Children were recruited through flyers sent to schools and advertisements on social media.”

We have also added the following paragraph in the **Control analyses** subsection (p. 13):

“Finally, although 5- and 8-year-olds did not differ with respect to either in-scanner motion (see **Methods**) or standardized IQ (see above), it remains possible that differences between groups in terms of motion or general cognitive functioning (i.e., IQ) might have affected our results. To exclude these possibilities, we performed a multiple linear regression analysis (with intercept) using the six head motion parameters and IQ as independent variables, as well as the decoding accuracy of each participant as a dependent variable. The predicted value associated with the independent variables was subtracted from the original decoding accuracy and was used as a new decoding accuracy. The group-level statistical significance was evaluated in the same manner as described above. Again, this analysis revealed very similar results, with significant between-age decoding accuracy in the bilateral PreCG and higher between-format decoding accuracy for 5-year-olds in the right IPL (**Figures S11, S12**). Therefore, differences between children’s in-scanner motion or IQ did not appear to have influenced our results.”

**Comment 1-16:** Methods, l.510-520: I do not understand how anatomical differences between children older than 5- to 8-years and adults relate to the current sample. Please clarify.

**Response 1-16:** We mentioned this potential issue because we used a standard adult Montreal Neurological Institute (MNI) space as a target for image normalization of 5- and 8-year-olds. This is a common practice in developmental cognitive neuroscience (Cantlon et al., J Cogn Neurosci, 2009; Perrachione et al., Neuron 2016). However, if the anatomical difference between children and adults is very large, it might affect the quality of normalization preprocessing. Previous studies cited in this paragraph (Kang et al., NeuroImage 2003; Szaflarski et al. NeuroImage 2012) have reported that it is not the case. Szaflarski et al. (2012) recruited children enrolled at ages 5, 6, 7 and examined those children annually for up to 10 years. Those MRI data with different ages were normalized into the same adult standard space. Kang et al. (2003) transformed 7- to 8-year-olds and adults' MRI data into the adult standard space. Both previous studies have indicated that normalizing children’s MRI data into adult standard space is valid. In our previous studies, we have also used the same normalization method for 8-year-olds (Girard et al., NPJ Sci Learn 2021; Girard et al., Psychol Sci 2022).

To clarify this point, we added the following text in the **fMRI data preprocessing** subsection of the Methods (p. 23):

“A standard practice in developmental cognitive neuroscience is to normalize functional images into the same adult stereotaxic space [[4,10,31]](https://paperpile.com/c/lxwFL4/LtCDA+Udmdr+q92RH). However, it is worth considering the possibility that anatomical differences between children and adults might affect the quality of the normalization.”

We also modified a sentence in the **fMRI data preprocessing** subsection as follows (p. 23):

“Studies have found that anatomical differences between children from 5 to 8 and adults are small enough that they are beyond the resolution of fMRI experiments [50,51].”

**Comment 1-17:** References: Formatting is inconsistent. Please double-check.

**Response 1-17:** We thank the reviewer for the comment. We set the citation style to that of PLOS Biology.

**Reviewer #2:**

**Comment 2-1:** I only have one question which is, regarding the adaptation paradigm, isn't it more traditional to model the deviant trials as events, rather than model the entire block? Could the authors expand on this methodological decision and briefly discuss the potential impact on results? This question is not critical to the acceptance of the paper in my opinion.

**Response 2-1:** We thank the reviewer for their positive assessment of the manuscript and for raising this point. We acknowledge that the current adaptation paradigm (adapted from Perrachione et al., Neuron, 2016) differs from previous paradigms used in the numerical cognition literature, mainly because it focuses on the adaptation period rather than the rebound activity. We made that choice because such block adaptation paradigms tend to be powerful as they capture larger adaptation effects than even-related designs (Grill-Spector et al., Trends Cogn Sci 2006), which is critical in pediatric neuroimaging. Importantly, a previous study reported that adaptation-related decreases of activity and rebound activity capture similar processes in the parietal cortex (Kersey and Cantlon, JNS 2017). Thus, this task appeared suitable for the current study.

In response to the reviewer’s comment, we added the following paragraph in the **Adaptation task** subsection of the Methods (p. 21):

“This task differs from typical adaptation designs used in the numerical cognition literature in that we focused here on the decrease of activity associated with the adaptation period instead of the ‘rebound’ activity that would be associated with a deviant presented after this adaptation period [[9]](https://paperpile.com/c/lxwFL4/qco1p). Although adaptation-related decreases in activity and rebound activity capture similar processes (including activity associated with numerosity processing in the IPS; [[4]](https://paperpile.com/c/lxwFL4/q92RH)), focusing on the adaptation period relies on more powerful block designs that typically capture a larger adaptation effect than that observed in event-related design [[48]](https://paperpile.com/c/lxwFL4/XuGA). This is particularly desirable when conducting relatively short experiments with young children.”

We also added the following citation:

“Grill-Spector K, Henson R, Martin A. Repetition and the brain: neural models of stimulus-specific effects. Trends Cogn Sci. 2006;10: 14–23. doi:10.1016/j.tics.2005.11.006”

In addition to the changes made based on the reviewers’ responses, we also made the following changes in the new manuscript:

We moved behavioral results of IQ tests from the **Methods** section to the **Result** section (p. 6):

“**Child measures**

To characterize the cognitive and mathematical skills of the samples, children’s intelligence quotient (IQ) and numerical skills were assessed outside of the scanner using standardized (age-normalized) batteries (see **Methods**). Average IQ, as measured by the NEMI-2 [[26]](https://paperpile.com/c/lxwFL4/ancaP), was in the normal range in each age group. Specifically, 5-year-olds had an average IQ of 109 (standard deviation [SD] = 15, range = 76–146) while 8-year-olds had an average IQ of 112 (SD = 11, range = 83–135). There was no difference in standardized IQ between the two groups (Wilcoxon rank-sum test, *p* = 0.18).”

**Reference:**

- Bulthé, J., De Smedt, B., & de Beeck, H. O. (2014). Format-dependent representations of symbolic and non-symbolic numbers in the human cortex as revealed by multi-voxel pattern analyses. NeuroImage, 87, 311-322.

- Cantlon, J. F., Libertus, M. E., Pinel, P., Dehaene, S., Brannon, E. M., & Pelphrey, K. A. (2009). The neural development of an abstract concept of number. Journal of cognitive neuroscience, 21(11), 2217-2229.

- Castaldi, E., Piazza, M., Dehaene, S., Vignaud, A., & Eger, E. (2019). Attentional amplification of neural codes for number independent of other quantities along the dorsal visual stream. Elife, 8, e45160.

- Cieslik, E. C., Zilles, K., Caspers, S., Roski, C., Kellermann, T. S., Jakobs, O., ... & Eickhoff, S. B. (2013). Is there “one” DLPFC in cognitive action control? Evidence for heterogeneity from co-activation-based parcellation. *Cerebral cortex*, *23*(11), 2677-2689.

- Eger, E., Michel, V., Thirion, B., Amadon, A., Dehaene, S., & Kleinschmidt, A. (2009). Deciphering cortical number coding from human brain activity patterns. Current Biology, 19(19), 1608-1615.

- Geary, D. C., Hoard, M. K., Nugent, L., & Bailey, D. H. (2012). Mathematical cognition deficits in children with learning disabilities and persistent low achievement: a five-year prospective study. *Journal of educational psychology*, *104*(1), 206.

- Girard, C., Bastelica, T., Léone, J., Epinat-Duclos, J., Longo, L., & Prado, J. (2022). Nurturing the mathematical brain: home numeracy practices are associated with children’s neural responses to Arabic numerals. Psychological Science, 33(2), 196-211.

- Girard, C., Bastelica, T., Léone, J., Epinat-Duclos, J., Longo, L., & Prado, J. (2021). Nurturing the reading brain: home literacy practices are associated with children’s neural response to printed words through vocabulary skills. NPJ science of learning, 6(1), 1-11.

- Harvey, B. M., & Dumoulin, S. O. (2017). A network of topographic numerosity maps in human association cortex. Nature Human Behaviour, 1(2), 1-9.

- Kang, H. C., Burgund, E. D., Lugar, H. M., Petersen, S. E., & Schlaggar, B. L. (2003). Comparison of functional activation foci in children and adults using a common stereotactic space. Neuroimage, 19(1), 16-28.

- Kersey, A. J., & Cantlon, J. F. (2017). Neural tuning to numerosity relates to perceptual tuning in 3–6-year-old children. Journal of Neuroscience, 37(3), 512-522.

- Knops, A., Thirion, B., Hubbard, E. M., Michel, V., & Dehaene, S. (2009). Recruitment of an area involved in eye movements during mental arithmetic. Science, 324(5934), 1583-1585.

- Núñez, R. E. (2017). Is there really an evolved capacity for number?. Trends in cognitive sciences, 21(6), 409-424.

- Perrachione, T. K., Del Tufo, S. N., Winter, R., Murtagh, J., Cyr, A., Chang, P., ... & Gabrieli, J. D. (2016). Dysfunction of rapid neural adaptation in dyslexia. Neuron, 92(6), 1383-1397.

- Piazza, M., Pica, P., Izard, V., Spelke, E. S., & Dehaene, S. (2013). Education enhances the acuity of the nonverbal approximate number system. Psychological science, 24(6), 1037-1043.

- Szaflarski, J. P., Altaye, M., Rajagopal, A., Eaton, K., Meng, X., Plante, E., & Holland, S. K. (2012). A 10-year longitudinal fMRI study of narrative comprehension in children and adolescents. Neuroimage, 63(3), 1188-1195.
