# Supplementary material for: Cortical representations of numbers and nonsymbolic quantities expand and segregate in children from 5 to 8 years of age
Source: PLoS Biol. 2023 Jan 5;21(1):e3001935. doi: 10.1371/journal.pbio.3001935 (PMC9815645; doi:10.1371/journal.pbio.3001935)
Supplement: Attachment — Submitted filename: Predys58Decoding_PB_Response_Rev2_Ver05.docx [file pbio.3001935.s024.docx]

We greatly appreciate the reviewers’ time and effort in reviewing our manuscript and providing helpful comments. Based on these comments, we have revised the original manuscript. Please find our responses to the comments below, along with the related changes in the revised manuscript.

**Reviewer #1:**

**Comment 1-1:** (1) Lack of neurobiological validity: A searchlight decoder is agnostic in terms of neurobiological validity. It just goes through each voxel and tries to solve a given decoding problem. This is why the current finding that visual numerosity is represented in auditory and body motor cortex requires neurobiological validation. But even the most powerful ultra-high-field fMRI study by Harvey et al. 2017 mentioned by the authors did not detect any visual numerosity representations in auditory and body motor cortices. Let alone any direct neuronal recording work (Nieder 2016). I find this really problematic because fMRI is prone to false-positive findings.

**Response 1-1:** We appreciate the reviewer’s point that searchlight decoding is agnostic in terms of neurobiological validity. In fact, this claim is not specific to searchlight decoding. The same criticism could be made of any sort of voxelwise neuroimaging studies, including those employing mass-univariate analyses (which essentially use a GLM to go through every single voxel in the brain). Clearly our study is not unique in using searchlight decoding, as shown by a large number of studies that have used it to address neurobiological questions (e.g., Kriegeskorte et al., PNAS 2006; Eger et al., Curr Biol 2009; Bulthé et al., NeuroImage 2014; Castaldi et al., NeuroImage 2016; Pennock et al., HBM 2021). In our opinion, the main advantage of searchlight decoding is precisely that it does not have any neurobiological constraint, allowing one to discover relations that would not have been found otherwise (by focusing on specific site or regions of interest). We believe that the field has made tremendous advances in addressing the issue of multiple comparisons in fMRI studies and our study uses what we think is probably the most adequate way to control for these false positives (i.e., non-parametric correction)

We also entirely agree with the reviewer that it could be surprising to find auditory or motor regions supporting visual numerosity processing. But again, we do not claim that *all* brain regions reported in Figure 2 process numerical quantity. This is why we acknowledge in the Discussion section that “even passively processing symbolic and non-symbolic quantity might involve a number of cognitive processes that are inherent to the processing of these stimuli (e.g., working-memory) and are therefore difficult (if not impossible) to control for.” In our main analysis of between-format decoding (Figure 4), we did not find significant decoding in auditory or body motor cortices, but instead in the right parietal cortex. Also, additional analysis of between-format decoding of digits and letters showed significant decoding in the bilateral fusiform gyri (Figure S8), a region known for its relation to the identification of both words and numerals from low-level visual features (Cohen et al., Brain 2002; Shum et al., JNS 2013). We do think that our critical findings support the view that our method has neurological validity.

**Comment 1-2:** (2) Lack of statistical explicitness and transparency: Given the moderate decoding accuracy reported here (in the range of 45-60%) I consider it as very important to make whole-brain voxel-wise decoding accuracy maps available to the reader (as I have asked in my previous review). Right now, it is impossible to determine which voxels contributed most strongly to the maximum decoding accuracy. The p-value maps buried in the Supplement do not contain this crucial information. In addition, having a closer look at these p-value maps (Fig.S1S2), I find it very concerning to see that p-values are highest in the canonical frontoparietal number system and lowest in frontal and occipital poles!?

**Response 1-2:** We apologize for our misunderstanding in the previous round of review. We had included the p-value maps based on the reviewer’s request to provide a statistical map of decoding accuracy. We have now uploaded the whole-brain voxel-wise decoding accuracy maps in the repository (“RawDecAcc.zip” in Zenodo repository; https://doi.org/10.5281/zenodo.7285545). We believe we are in fact very transparent regarding our results, supplementary figures and materials shared on Zenodo. As can be seen online, we have provided extensive data supporting our findings.

**Comment 1-3:** (3) Liberal multiple comparison correction: It is unclear to me why the authors stick to their liberal cluster-forming threshold of p < 0.005 instead of reanalyzing the data using the the well-established threshold of p < 0.001 (see e.g. Cavdaroglu & Knops 2019). To ensure replicability by other labs, I see it as essential to provide direct empirical evidence based on the present dataset that the permutation test chosen here robustly removes false-positive activation across cluster-forming thresholds.

**Response 1-3:** We did not change our initial voxel-level threshold because we did not find any statistical argument provided by either the reviewer or the literature that our choice was invalid in the context of the non-parametric control method we used (Eklund, PNAS 2016). Nonetheless, we have now performed a full set of additional analyses using a voxel-level threshold of *p* < 0.001 instead of p < 0.005. These new analyses largely replicate our main findings, supporting simulations showing that non-parametric control methods are much more robust to changes in voxel-level thresholds than parametric methods (Eklund, PNAS 2016).

The results of these new analyses are shown in Supplementary Figure 13 and described in a new paragraph on p. 13. There, we state:

“Second, we recalculated the significance of clusters in decoding accuracy maps using a voxel-level threshold of *p* < 0.001 instead of *p* < 0.005. Results were again very similar to our main findings. As shown in **Figure S13**, we still found (1) greater within-format decoding accuracy in 8-year-olds than in 5-year-olds in frontal, parietal, and occipital brain regions, (2) significant within-format decoding accuracy across groups in the prefrontal and occipital cortex, and most importantly (3) significant between-format decoding in the right IPL for 5-year-olds, but not 8-year-olds. Altogether, these results indicate that the present findings did not hinge upon a specific cross-validation method or a specific thresholding.”

We hope that this new set of analyses will provide more confidence in our results. Note that we agree that the voxel-wise threshold of p < 0.001 is widely used in the literature, but it is also important to keep in mind that this convention largely comes from limitations placed upon parametric control methods studies (which also largely employed mass-univariate rather than multivariate analyses). Our initial voxel-level threshold of p < 0.005 in the present study was chosen to be consistent with our most recent study using multivariate representational similarity analysis (Bhatia et al., Cereb Cortex 2022). This is now made clear on p. 25 of the revised manuscript, where we state:

“In line with our most recent fMRI study using multivariate analyses [[15]](https://paperpile.com/c/6mnHwS/nWda), the statistical threshold was set at *p* < 0.005 for the voxel level and at *p* < 0.05 for the cluster level. Additional control analyses, however, use a voxel level of *p* < 0.001 to ensure that this parameter does not critically affect our conclusions (**Figure S13**).”

**Comment 1-4:** (4) Statistical power unknown: Sample size selection is not guided by an a priori power analysis so that actual statistical power is not known.

**Response 1-4:** The reviewer is correct that our sample size for the decoding analysis was not guided by an a priori power analysis. Rather, our sample size was mainly constrained by available resources, as the data underlying the analyses reported in the paper are also part of a large project notably investigating the home learning environment of these children (Girard et al., npj Sci Learn, 2021; Girard et al., Psychol Sci 2022). We still note that our sample sizes (N = 89) are a significant improvement from previous studies on a similar topic with a similar age group (N = 14 in Cantlon et al. J Cogn Neurosci 2009; N = 34 in Kersey & Cantlon, JNS 2017; N = 36 in Hyde et al. NeuroImage 2010; N = 36 in Izard et al., PLOS Biol 2008). Nonetheless, performing a power analysis for our study would have been quite challenging for two reasons. First, power analyses are still very rare in decoding studies (Hebart & Baker, NeuroImage 2018). The main reason is that there is no widely accepted measure of what an effect size would be in such analyses, in contrast to mass-univariate analyses in which a Cohen’s d can potentially be derived from differences in beta estimates. Second, setting aside this major issue, it would still have been quite difficult to have an estimate of the anticipated effect size, considering the scarcity of previous neuroimaging data on children from the age range, the fact that none of these studies investigated multivariate decoding, and the general lack of transparency in these studies in general regarding what could be considered an effect size. For these reasons, it is unclear to us how a power analysis could have been conducted here. However, we certainly believe that our findings may provide important information for guiding future studies. This is why we provide in the paper all of the statistical and accuracy maps on a public repository. We hope future studies will use such information to allow power analyses to be performed.

**Comment 1-5:** (5) Behavioral relevance: The present work does not speak to the important question of whether the reported neural data explain behavior (Krakauer et al. 2017). It remains open whether neural representation metrics relate to individual mathematical test performance scores.

**Response 1-5:** We completely agree with the reviewer’s comment and do not think we said otherwise in the manuscript (we simply said that such brain-behavior correlation studies will require a much larger number of participants, see p. 19 and 20). We believe future studies should examine the relation between format-invariance of quantity representation and individual math performance.

**Comment 1-6:** (6) Mismatch between stimuli: Dots did not match digits in the current task (6-60 vs. 1-8). Comparing these conditions would thus be comparing apples and oranges. Accordingly, the authors justifiably did not directly compare dots and digits. But this also means that (for experimental reasons) their data do not support their interpretations about the association between symbolic and non-symbolic quantity.

**Response 1-6:** Yes, we agree with the reviewer that our stimuli do not match in term of quantity. But, as we explained in the previous rebuttal letter, the goal of the study was not to evaluate whether exact symbolic processing (the digit 4) relates to exact non-symbolic processing (4 dots). The goal of the study was to examine whether symbolic processing relates to an approximate sense of numerosity, which can only be carried by larger sets of dots. This is addressed in p. 20 of the discussion:

“Finally, the main goal of the present study was to test the influential hypothesis that learning symbolic numbers builds on an approximate sense of quantity that is associated with the ANS [5]. To elicit ANS processing, we adapted our non-symbolic stimuli from previous tasks in which participants are typically presented with relatively large sets of dots that cannot be enumerated (thereby preventing exact symbolic processing). However, because young children in kindergarten do not master the symbolic place-value notation, our symbolic stimuli did not include double-digit numbers and were restricted to Arabic numerals. In other words, symbolic and non-symbolic stimuli did not represent the same quantities. Therefore, although our results inform about the relation between the neural representations of Arabic numerals and approximate non-symbolic quantities in young children, our conclusions might not extend to the relation between exact symbolic and exact non-symbolic quantities.”

**Comment 1-7:** (7) Selection bias: I find it severely misleading to label an average IQ of 109 or even 112 as "slightly above the norm". Instead, families with above-average intelligence were significantly overrepresented here. This problem is aggravated in my view by the fact that the 49 excluded children (about 40% of the entire sample) were less intelligent (p = 0.06) than the 89 children who were retained for MRI scanning. As a consequence, I see no empirical basis for generalizing the reported findings to general population.

**Response 1-7:** We were fairly inclusive in our recruiting strategy, only excluding three children who had an IQ lower than the 2.5th percentile (putting them in the mental retardation range). We excluded these children because the tasks and scanner environment would have been very challenging for them. Nonetheless, it is true that the mean IQ in both groups (i.e., children included in the final analyses) can be considered high average, with a score of 109 for 5-year-olds and 112 for 8-year-olds. Note that these numbers are often found in task-fMRI studies with young children. For example, mean IQ was 107 and 123 in Superkar et al. (PNAS 2013); 114.3 and 116.8 in Qin et al. (Nat Neurosci 2014); 119.6 in Perrachione et al. (Neuron, 2016); and 107.5, 111.1, and 109.7 in Suárez-Pellicioni et al. (Sci Data, 2019). Therefore, our target population does not deviate from previous studies. In our opinion, the most important criterion is whether IQ in itself can explain our results, which we refuted in control analyses (see first paragraph in p. 13).

In line with the editor’s suggestion, we now include a new paragraph in the discussion in which we address this issue. On p. 18 and 19, the paragraph reads:

“Second, it is always important to wonder whether our participants are representative of the general population. To ensure the representativity of our samples, we recruited children from various areas within the Lyon metropolitan area. We were also inclusive of children with relatively low cognitive functioning, only excluding children with mental retardation (based on an IQ lower than the 2.5^th^ percentile) because they would not have been able to adequately complete the tasks. Overall, the mean IQ in both groups can be considered high average, with a score of 109 for 5-year-olds and 112 for 8-year-olds. Such high average numbers are relatively common in the developmental cognitive literature [[31,42]](https://paperpile.com/c/6mnHwS/8xnD+926Pu). They might be inevitable considering (1) the challenges raised by recruiting diverse samples in lab-based developmental studies and (2) the need to remove from the fMRI data analyses participants with high motion and poor task performance (both of these being potentially related to levels of attention and general cognitive functioning). In our view, the most critical aspect for generalizability is to ensure that none of the findings are related to individual differences in cognitive function, which we ensured in the present study (see control analyses above). Therefore, although future studies might extend our findings to children with lower cognitive functioning, we believe that our findings remain generalizable to the population.”

Note that, in addition to the changes made based on the reviewers’ responses, we also made two additional changes.

First, we made some minor stylistic changes in the first paragraph of the introduction:

“Prior studies suggest that brain sensitivity to approximate non-symbolic quantities precedes the acquisition of number symbols in children[[1,2]](https://paperpile.com/c/6mnHwS/FMs1P+9xEy3), with early neural processing of approximate non-symbolic quantities reported around the intraparietal sulcus (IPS) [[1–4]](https://paperpile.com/c/6mnHwS/FMs1P+9xEy3+KiZkc+avD0N). This is consistent with the idea that an early-developing ‘approximate number system’ (ANS) [[5]](https://paperpile.com/c/6mnHwS/X11L6) may scaffold the development of symbolic numerical knowledge.”

Second, in line with the changes made for explaining our additional analyses (Figure S13), we added the following explanations to the Control analyses subsection:

“Finally, decoding accuracy might depend (to some extent) on the choice of analysis parameters, such as the type of cross-validation procedure and the voxel-level threshold that provides the basis for cluster-level correction [[29]](https://paperpile.com/c/6mnHwS/ysU2). To evaluate to what extent our results were sensitive to these choices, we performed additional analyses using a different type of cross-validation procedure and a different voxel-level threshold. First, we computed decoding accuracy maps using 10-fold cross-validation instead of LOOCV [[30]](https://paperpile.com/c/6mnHwS/gN3oL). As shown in **Figures S11, S12,** we obtained results that were very similar to our main findings. Notably, we found significant between-format decoding accuracy in the right IPL for 5-year-olds, but not 8-year-olds.”

Related the above paragraph, we added the following citation:

“[Botvinik-Nezer R, Holzmeister F, Camerer CF, Dreber A, Huber J, Johannesson M, et al. Variability in the analysis of a single neuroimaging dataset by many teams. Nature. 2020;582: 84–88.](http://paperpile.com/b/6mnHwS/ysU2)”

**References**

Bhatia, P., Longo, L., Chesnokova, H., & Prado, J. (2022). Neural Representations of Absolute and Relative Magnitudes in Symbolic and Nonsymbolic Formats. Cerebral Cortex.

Bulthé, J., De Smedt, B., & de Beeck, H. O. (2014). Format-dependent representations of symbolic and non-symbolic numbers in the human cortex as revealed by multi-voxel pattern analyses. NeuroImage, 87, 311-322.

Cantlon, J. F., Libertus, M. E., Pinel, P., Dehaene, S., Brannon, E. M., & Pelphrey, K. A. (2009). The neural development of an abstract concept of number. Journal of cognitive neuroscience, 21(11), 2217-2229.

Castaldi, E., Aagten-Murphy, D., Tosetti, M., Burr, D., & Morrone, M. C. (2016). Effects of adaptation on numerosity decoding in the human brain. *NeuroImage*, *143*, 364-377.

Cohen, L., & Dehaene, S. (2004). Specialization within the ventral stream: the case for the visual word form area. *Neuroimage*, *22*(1), 466-476.

Eger, E., Michel, V., Thirion, B., Amadon, A., Dehaene, S., & Kleinschmidt, A. (2009). Deciphering cortical number coding from human brain activity patterns. Current Biology, 19(19), 1608-1615.

Eklund, A., Nichols, T. E., & Knutsson, H. (2016). Cluster failure: Why fMRI inferences for spatial extent have inflated false-positive rates. Proceedings of the national academy of sciences, 113(28), 7900-7905.

Girard, C., Bastelica, T., Léone, J., Epinat-Duclos, J., Longo, L., & Prado, J. (2021). Nurturing the reading brain: home literacy practices are associated with children’s neural response to printed words through vocabulary skills. NPJ science of learning, 6(1), 1-11.

Girard, C., Bastelica, T., Léone, J., Epinat-Duclos, J., Longo, L., & Prado, J. (2022). Nurturing the mathematical brain: home numeracy practices are associated with children’s neural responses to Arabic numerals. Psychological Science, 33(2), 196-211.

Kersey, A. J., & Cantlon, J. F. (2017). Neural tuning to numerosity relates to perceptual tuning in 3–6-year-old children. Journal of Neuroscience, 37(3), 512-522.

Kriegeskorte, N., Goebel, R., & Bandettini, P. (2006). Information-based functional brain mapping. Proceedings of the National Academy of Sciences, 103(10), 3863-3868.

Hebart, M. N., & Baker, C. I. (2018). Deconstructing multivariate decoding for the study of brain function. Neuroimage, 180, 4-18.

Hyde, D. C., Boas, D. A., Blair, C., & Carey, S. (2010). Near-infrared spectroscopy shows right parietal specialization for number in pre-verbal infants. Neuroimage, 53(2), 647-652.

Izard, V., Dehaene-Lambertz, G., & Dehaene, S. (2008). Distinct cerebral pathways for object identity and number in human infants. PLoS biology, 6(2), e11.

Pennock, I. M. L., Schmidt, T. T., Zorbek, D., & Blankenburg, F. (2021). Representation of visual numerosity information during working memory in humans: An fMRI decoding study. Human Brain Mapping, 42(9), 2778-2789.

Perrachione, T. K., Del Tufo, S. N., Winter, R., Murtagh, J., Cyr, A., Chang, P., ... & Gabrieli, J. D. (2016). Dysfunction of rapid neural adaptation in dyslexia. Neuron, 92(6), 1383-1397.

Qin, S., Cho, S., Chen, T., Rosenberg-Lee, M., Geary, D. C., & Menon, V. (2014). Hippocampal-neocortical functional reorganization underlies children's cognitive development. Nature neuroscience, 17(9), 1263-1269.

Shum, J., Hermes, D., Foster, B. L., Dastjerdi, M., Rangarajan, V., Winawer, J., ... & Parvizi, J. (2013). A brain area for visual numerals. Journal of Neuroscience, 33(16), 6709-6715.

Suárez-Pellicioni, M., Fuchs, L., & Booth, J. R. (2019). Temporo-frontal activation during phonological processing predicts gains in arithmetic facts in young children. Developmental Cognitive Neuroscience, 40, 100735.

Supekar, K., Swigart, A. G., Tenison, C., Jolles, D. D., Rosenberg-Lee, M., Fuchs, L., & Menon, V. (2013). Neural predictors of individual differences in response to math tutoring in primary-grade school children. *Proceedings of the National Academy of Sciences*, *110*(20), 8230-8235.
